# Solution Structure of CCP Modules 10–12 Illuminates Functional Architecture of the Complement Regulator, Factor H

**DOI:** 10.1016/j.jmb.2012.09.013

**Published:** 2012-12-14

**Authors:** Elisavet Makou, Haydyn D.T. Mertens, Mateusz Maciejewski, Dinesh C. Soares, Ilias Matis, Christoph Q. Schmidt, Andrew P. Herbert, Dmitri I. Svergun, Paul N. Barlow

**Affiliations:** 1 Schools of Chemistry and Biological Sciences, University of Edinburgh, West Mains Road, Edinburgh EH9 3JJ, UK; 2 European Molecular Biology Laboratory Hamburg Outstation, c/o Deutsches Elektronen‐Synchrotron, Notkestrasse 85, 22603 Hamburg, Germany; 3 Medical Genetics Section, Molecular Medicine Centre, Institute of Genetics and Molecular Medicine, University of Edinburgh Western General Hospital, Crewe Road South, Edinburgh EH4 2XU, UK

**Keywords:** CCP, complement control protein, CR1, complement receptor type 1, DAF, decay accelerating factor, FH, factor H, EOM, ensemble optimization method, HSQC, heteronuclear single quantum coherence, MCP, membrane cofactor protein, NOE, nuclear Overhauser enhancement, SAXS, small-angle X-ray scattering, TOCSY, total correlated spectroscopy, protein NMR, protein domains, complement system, small-angle X-ray scattering, regulators of complement activation

## Abstract

The 155-kDa plasma glycoprotein factor H (FH), which consists of 20 complement control protein (CCP) modules, protects self-tissue but not foreign organisms from damage by the complement cascade. Protection is achieved by selective engagement of FH, via CCPs 1–4, CCPs 6–8 and CCPs 19–20, with polyanion-rich host surfaces that bear covalently attached, activation-specific, fragments of complement component C3. The role of intervening CCPs 9–18 in this process is obscured by lack of structural knowledge. We have concatenated new high-resolution solution structures of overlapping recombinant CCP pairs, 10–11 and 11–12, to form a three-dimensional structure of CCPs 10–12 and validated it by small-angle X-ray scattering of the recombinant triple‐module fragment. Superimposing CCP 12 of this 10–12 structure with CCP 12 from the previously solved CCP 12–13 structure yielded an S-shaped structure for CCPs 10–13 in which modules are tilted by 80–110° with respect to immediate neighbors, but the bend between CCPs 10 and 11 is counter to the arc traced by CCPs 11–13. Including this four-CCP structure in interpretation of scattering data for the longer recombinant segments, CCPs 10–15 and 8–15, implied flexible attachment of CCPs 8 and 9 to CCP 10 but compact and intimate arrangements of CCP 14 with CCPs 12, 13 and 15. Taken together with difficulties in recombinant production of module pairs 13–14 and 14–15, the aberrant structure of CCP 13 and the variability of 13–14 linker sequences among orthologues, a structural dependency of CCP 14 on its neighbors is suggested; this has implications for the FH mechanism.

## Graphical abstract

**Graphical abstract:**

**Figure:**
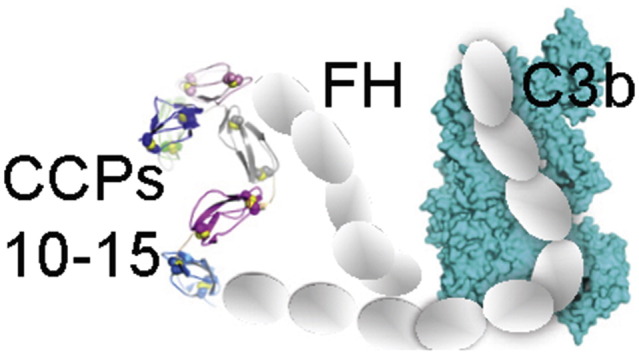

**Highlights:**

► The 20-CCP‐module human protein FH prevents complement-mediated tissue damage. ► NMR structures of CCPs 10–11 and 11–12 suggest that this region enhances flexional strength of FH. ► Concatenating bi-modules helps interpret small‐angle X‐ray scattering data, revealing highly compacted arrangement of CCPs 13, 14 and 15. ► Apparent structural dependency of CCP 14 on neighbors could provide a switch between ordered and flexible FH architectures.

## Introduction

The activated complement system[1], [2], [3] assists in clearance of pathogens in addition to debris from diseased, damaged, dead or dying cells. Hydrolysis of a thioester linkage in complement component C3, yielding C3(H_2_O), is the rare, spontaneous but ubiquitous initiating event of the “alternative” pathway of the complement cascade.^4^ C3(H_2_O) collaborates with proenzyme factor B and proteolytic factor D to create the convertase complex C3(H_2_O).Bb that converts C3 to C3a (an anaphylatoxin) and C3b. C3b is able to rapidly attach covalently to any nearby surface and to form C3b.Bb [a structural and functional analogue of C3(H_2_O).Bb] that, in a positive-feedback loop,^5^ converts additional C3 to C3b. Particles become coated (“opsonised”) with C3b and are then subject to immune clearance. Moreover, C3b participates in both “classical” and “lectin” pathways; all three pathways feed into the terminal pathway that releases the anaphylatoxin C5a and culminates in the assembly of potentially cytolytic pores.

Distinction by the alternative pathway between self and nonself is not driven by discriminatory covalent attachment of C3b.[4], [6] Rather, it is accomplished mainly by selective action of factor H (FH), complement receptor type 1 (CR1), membrane cofactor protein (MCP) and decay accelerating factor (DAF).^7^ Either through being host-membrane bound (CR1, DAF or MCP) or (in the case of FH) via selective association with host-cell‐specific markers,^8^ these regulators prevent amplification of C3b on self-surfaces. They (DAF, CR1 and FH) perturb formation and stability of C3b.Bb or (CR1, FH and MCP) recruit factor I to cleave C3b, removing it from the amplification loop that continues to operate on, for example, a bacterium. The product, iC3b, cannot form a complex with Bb but remains an opsonin and a ligand for receptors on immune cells.^9^

The abundant^10^ plasma regulator FH (155 kDa, 1213 residues)[11], [12] fails to adequately protect host tissues from complement-mediated damage over the lifetimes of many individuals who possess *CFH* polymorphisms or mutations.^13^ Age-related macular degeneration is a prominent example of a disease arising from such a deficiency.^14^ To explain the molecular bases for these disorders, it is essential to understand why wild-type FH is significantly more active on healthy self-cells than on diseased or foreign ones.^15^ Moreover, as a self-tissue‐specific regulator of C3 activation, knowledge of the structure–function relationships of FH^16^ could inspire design of therapeutic complement inhibitors useful in the wide range of conditions where complement plays a damaging role.[13], [17], [18], [19]

**Fig. 1:**
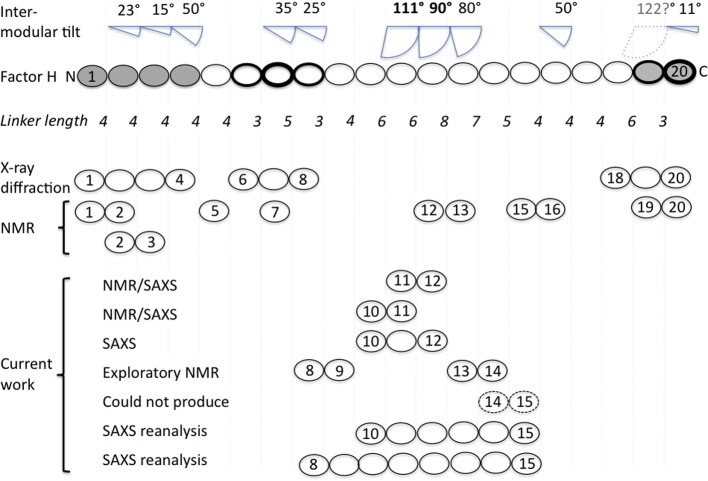
Gaps in structural knowledge of human complement FH. Drawn as ovals, CCPs are shaded to indicate C3b-binding regions or emboldened to signify GAG/sialic acid-recognition sites (CCPs 7 and 20 being the most strongly implicated). The number of residues in each inter-modular linker is written beneath; above is a schematic representation of the tilt angle (away from linearity) of a CCP's long axis with respect to the long axis of the preceding CCP [including (bold) FH 10–11 and FH 11–12 tilts measured herein]. The tilt between CCPs 18 and 19 was 122° in the FH 18–20 crystal structure,^36^ but SAXS suggested a more extended arrangement in solution. Structures solved by NMR or X-ray crystallography of recombinant segments of FH are summarized in the central portion of the figure. “Current work” summarizes the recombinant constructs investigated and methods used for analysis.

FH consists entirely of 20 complement control protein (CCP) modules^20^ of between 51 and 62 residues each, connected by linking sequences of between 3 and 8 residues in length[21], [22] (Fig. 1). Successful crystallization of FH has not been reported. Current structural knowledge draws on data from small-angle X-ray scattering (SAXS), analytical ultracentrifugation and electron microscopy[23], [24], [25], [26] combined with high-resolution structures for short discontinuous segments of the protein.[27], [28], [29], [30], [31], [32], [33], [34], [35], [36]

The N-terminal CCPs 1–4 and the C-terminal CCPs 19–20 of FH bind C3b at adjacent, nonoverlapping sites.[34], [37], [38] Like full-length FH, a recombinant fragment consisting solely of CCPs 1–4 (i.e., FH 1–4) not only competes with binding of factor B to C3b in solution but also recruits factor I.[39], [40], [41], [42] In addition, FH has sites within CCPs 6–8 and CCPs 19–20 that interact with polyanionic self-surface markers and are critical for specificity.[37], [43], [44], [45], [46] Nearly all disease-linked sequence variations occur at these C3b-binding or polyanion-binding regions of FH^13^ in which CCPs adopt extended end-to-end arrangements connected by linkers of three or four residues. The central modules of FH (CCPs 9–18), on the other hand, do not contain strong binding sites for polyanions or C3b.^37^ Thus, tethering together the two C3b/polyanion-binding sites, CCPs 1–8 and CCPs 19–20, might produce an efficient biopharmaceutical.^47^ However, knowledge on the role of the intervening 10 modules of FH that are hypothesized to enhance the specificity of FH for self-surface protection is lacking.

This central region has long inter‐modular linkers (6, 6, 8, 7 and 5 residues for CCPs 10–11, 11–12, 12–13, 13–14 and 14–15, respectively) (Fig. 1), relatively small modules (51 residues in CCP 13), and accommodates seven of eight utilized N-glycosylation sites in FH.^48^ Initial notions that CCPs 9–18 merely form a flexible tether between C3b-binding and polyanion-recognition regions were disproved by studies of FH 10–15.^35^ This molecule failed to crystallize, but a SAXS-derived structural model guided by an NMR-derived FH 12–13 structure was created. The FH 10–15 construct forms a compact rather than flexible or elongated structure.

Here, we describe recombinant production of the remaining bi-modules (i.e., besides FH 12–13) from the FH 10–15 region and their structural investigation. While samples of FH 13–14 and FH 14–15, unusually for CCP bi-modules, resisted all attempts at preparation in our hands, structures of FH 10–11 and FH 11–12 were readily determined by NMR. A structure of FH 10–12, produced by combining these two high-resolution solution structures, was validated by SAXS. A subsequently constructed model of FH 10–13 allowed reinterpretation of SAXS data for FH 10–15 and FH 8–15. It seems that CCPs 13–15 are compacted, while CCP 14 appears unstable unless it is folded back along CCPs 12 and 13. These possibilities are discussed in terms of the mechanism of FH.

## Results

### FH 10–11 and FH 11–12, but not FH 13–14 or FH 14–15, yielded assignable NMR spectra

**Fig. 2:**
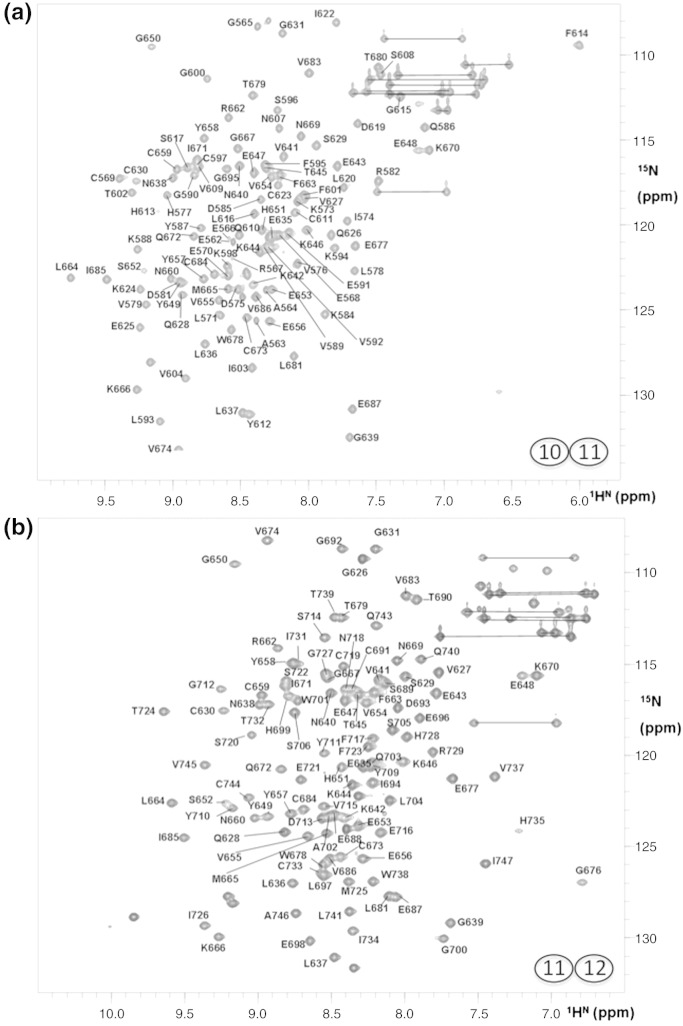
NMR data ([^1^H,^15^N]HSQC spectra) for FH 10–11 and FH 11–12. Spectra [(a) FH 10–11; (b) FH 11–12] were collected in 30 min on double-labeled samples (0.45 mM and 0.65 mM FH 10–11 and FH 11–12, respectively) at 25 °C in 20 mM potassium phosphate buffer (at pH 6.7 and 6.3 for FH 10–11 and FH 11–12, respectively).

We set out to investigate whether the large tilt angle (~ 80°) previously observed between CCPs 12 and 13^35^ is a consistent feature across the central segment of FH. Like FH 12–13, samples of FH 10–11 and FH 11–12 were straightforward to prepare. Moreover, they gave [^1^H,^15^N]heteronuclear single quantum coherence (HSQC) spectra of high quality with the expected numbers of well‐dispersed cross-peaks (Fig. 2a and b) for stably folded proteins.

Other overlapping bi-modules within the FH 10–15 region—FH 13–14 and FH 14–15—proved intractable. Recombinant FH 13–14 was prone to proteolysis and self-association; nevertheless, a ^15^N-labeled sample eluting as a monomeric species was obtained from a calibrated size-exclusion chromatography column and produced a single band, of the expected size, by sodium dodecyl sulfate–polyacrylamide gel electrophoresis. Unexpectedly, this FH 13–14 sample must have been improperly folded since, under a range of conditions, it yielded [^1^H,^15^N]HSQC spectra (Supplementary Fig. S1) dominated by overlapped, broad cross-peaks. An overlay of this spectrum with a [^1^H,^15^N]HSQC spectrum of FH 13 (previously expressed as an isolated module^35^) showed that only a few weak cross-peaks from compactly folded CCP 13 could be detected in the CCP 13–14 spectrum (Supplementary Fig. S1). In the case of FH 14–15, no sample suitable for NMR data collection could be prepared due to excessive propensity for proteolysis and oligomerization.

### Modules 10–12 do not form a highly elongated structure

**Fig. 3:**
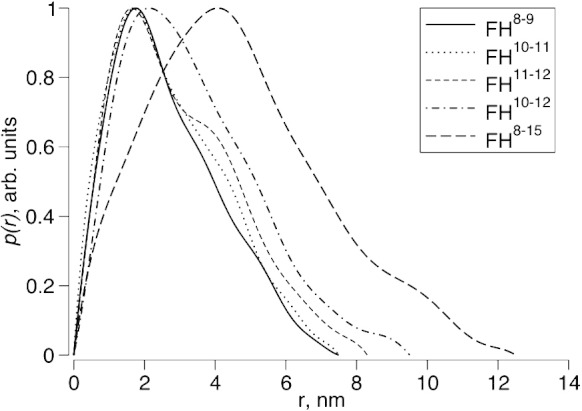
SAXS data for FH constructs: pairwise interatomic distance distributions, *p*(*r*), for the following constructs derived from FH, FH 8–9, FH 10–11, FH 11–12, FH 10–12 and FH 8–15.

SAXS curves were collected for FH 10–11, FH 11–12 and FH 10–12; neither FH 13–14 nor FH 14–15 samples were suitable. Analysis of parameters extracted from scattering profiles and real-space distance distribution functions, *p*(*r*), reveals monomers (Fig. 3 and Table 1) consistent with their elution volumes from size-exclusion chromatography columns. Bi-modules FH 10–11 and FH 11–12 yielded similar radii of gyration (*R*_g_) and maximum particle dimensions (*D*_max_), but FH 11–12 (*R*_g_ = 2.4 nm and *D*_max_ = 8.3 nm) was slightly more extended than FH 10–11 (*R*_g_ = 2.2 nm and *D*_max_ = 7.5 nm). The positively skewed *p*(*r*) functions of both bi-modules are characteristic of dumbbell-shaped structures; the first maximum represents a common average intra-module distance (~ 1.7 nm), and the second maximum represents an approximate average separation of the centers of mass of the two CCPs (~  3.8 nm). Studies of tri-modular FH 10–12 yielded only a modest 0.1‐ to 0.3‐nm increase in *R*_g_ and 0.8‐ to 1.2‐nm increase in *D*_max_ (Fig. 3 and Table 1). The *p*(*r*) function of FH 10–12 shows a shift in the position of the first maximum relative to that of bi-modules; this reflects an increase in effective cross-section upon addition of the third CCP. These observations clearly demonstrate that FH 10–12 does not have an elongated structure similar to FH 1–3^33^ or FH 6–8.^32^

**Table 1:** SAXS parameters for FH fragments

Construct	*R*_g_^autoRg^ (nm)	*R*_g_^GNOM^ (nm)	*D*_max_ (nm)	*V*_p_ (nm^3^)	MM_SAXS_ (kDa)	*V*_DAM_ (nm^3^)	MM_DAM_ (kDa)
FH 8–9 (13.7 kDa)	2.1 ± 0.1	2.2 ± 0.1	7.5 ± 0.5	21 ± 5	15 ± 5	23 ± 5	11 ± 5
FH 10–11 (13.9 kDa)	2.2 ± 0.1	2.2 ± 0.1	7.5 ± 0.5	22 ± 5	16 ± 5	21 ± 5	10 ± 5
FH 11–12 (14.1 kDa)	2.4 ± 0.1	2.3 ± 0.1	8.3 ± 0.5	25 ± 5	19 ± 5	25 ± 5	12 ± 5
FH 10–12 (20.6 kDa)	2.7 ± 0.1	2.7 ± 0.1	9.5 ± 0.5	33 ± 5	20 ± 5	47 ± 5	24 ± 5
FH 8–15 (54.7 kDa)	3.5 ± 0.1	3.7 ± 0.1	12.5 ± 0.5	92 ± 10	62 ± 5	93 ± 10	47 ± 5

*R*_g_^autoRg^ and *R*_g_^GNOM^ are the radii of gyration estimated from the SAXS data using the automated Guinier analysis routine and GNOM, respectively. *D*_max_, *V*_p_, *V*_DAM_, MM_SAXS_ and MM_DAM_ are the maximum particle dimension, hydrated particle volume, dummy-atom model total excluded volume, molecular mass estimated from *I*(0) and molecular mass estimated from the dummy-atom model volume, respectively. The data shown are averaged or merged and extrapolated to infinite dilution, using the SAXS profiles recorded at several concentrations. The molecular mass calculated from the sequence is shown in parentheses.

### Three-dimensional structures of FH 10–11 and FH 11–12

**Fig. 4:**
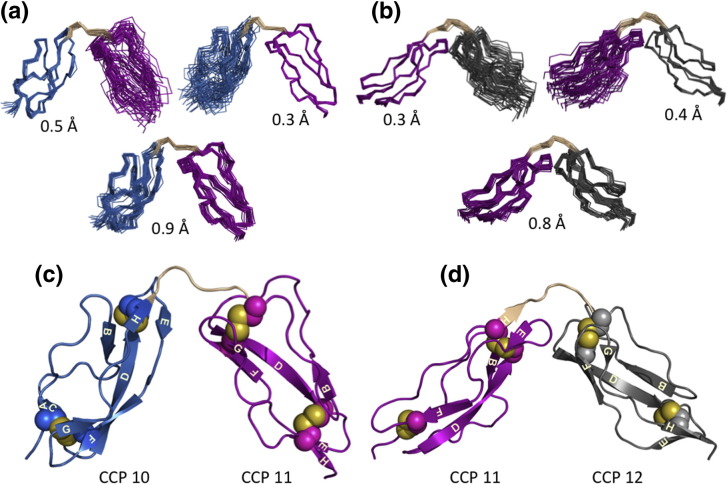
Solution (NOE-derived) structures of FH 10–11 and FH 11–12. (a) The 20 lowest-energy, water-minimized FH 10–11 structures, overlaid on backbone atoms of CCP 10 (blue), CCP 11 (magenta) or both modules CCP 10 and 11 (and linker; wheat ) (relevant rmsd values are shown). (b) As (a) except that NOE-derived structures of FH 11–12 (CCP 12 in gray) are overlaid. In the cartoon representations of (c) FH 10–11 and (d) FH 11–12, β‐strands identified by STRIDE were labeled A–H according to a convention whereby CCP modules have a maximum of eight β‐strands. Spheres represent cysteine side chains (sulfurs are colored yellow).

The NMR spectra (^13^C,^15^N) for FH 10–11 and 11–12 (Fig. 2a and b) enabled near-complete assignment of ^1^H, ^15^N and ^13^C (Table 2). Following manual partial assignment of ^15^N‐ and ^13^C-filtered nuclear Overhauser enhancement (NOE) spectroscopy spectra, we input a provisional list of NOE assignments into the first of seven CYANA cycles of structure calculations.^49^ CYANA-derived distance restraints were used for calculations of structures in CNS (*C*rystallography and *N*MR *S*ystem).^50^ The 20 lowest-energy CNS-derived structures converged well and were refined in explicit aqueous solvent (Fig. 4a and b; Table 2). Note (as discussed further below) that near-identical structures resulted from a calculation that incorporated both NOEs and SAXS-derived parameters.

**Table 2:** Statistics for solution structures of FH 10–11 and FH 11–12

Segment	FH 10–11	FH 11–12
Numbers of NOE-derived distance restraints		
Intraresidue, |*i* − *j*| ≤ 1	1405	1482
Medium range, 1 < |*i* − *j*| < 5	411	301
Long range, |*i* − *j*| ≥ 5	1391	1322
Total	3207	3105
Inter‐modular^a^	8	38
N-module to linker	62	89
C-module to linker	65	39
rmsd values (Å) (superimposition of ensemble over entire sequence)		
All atoms	1.17	1.05
Backbone atoms	0.91	0.84
rmsd values (Å) (superimposition of ensemble over N-terminal CCP)		
All atoms	0.79	0.73
Backbone atoms	0.50	0.33
rmsd values (Å) (superimposition of ensemble over C-terminal CCP)		
All atoms	0.74	0.72
Backbone atoms	0.33	0.43
Inter‐modular angles (°) [minimum–maximum (mean ± SD)]		
Skew	75–107 (89 ± 8)	149–170 (158 ± 4)
Twist	40–78 (57 ± 10)	59–87 (72 ± 7)
Tilt	111–128 (118 ± 5)	76–99 (88 ± 6)
Ramachandran assessment (%)		
Most favored	80.5	74.6
Additionally allowed	16.4	22.4
Generously allowed	1.3	1.7
Disallowed	1.8	1.3
Surface area buried between modules (Å^2^)^b^	358	642

a Module boundaries defined by the first and the last cysteine of each CCP module.

b This value is for the closest-to-mean structures in each ensembles (see Materials and Methods).

The β-strand-rich structures of CCPs 10, 11 and 12 (Fig. 4c and d) contain Cys(I)–Cys(III) and Cys(II)–Cys(IV) disulfides. The consensus Trp is buried in the cores of CCPs 11 and 12; a Leu occupies this position in CCP 10. STRIDE-identified^51^ β-strands were labeled (in Fig. 4c and d) according to a convention recognizing the occurrence of maximally eight β-strands (A–H), at conserved positions, within CCP structures.[29], [52] Slowly exchanging amide protons (Supplementary Fig. S2) coincided with either burial or engagement in hydrogen bonds; after 15 min of D_2_O exposure, the following numbers of HSQC cross-peaks remained: 2 in CCP 10 and 22 in CCP 11 (none in the linker) for FH 10–11, and 17 in CCP 11 and 9 in CCP 12 (1 in the linker) for FH 11–12. Although CCP 10 has more extensive β-strands, CCPs 11 and 12 have more slowly exchanging amides.

Structures of CCP 10 or 11 converged well when the ensemble of NOE-derived FH 10–11 structures was overlaid solely on either module; CCP 11 or 12 within the FH 11–12 ensemble likewise converged well on a per-module basis (Fig. 4a and b). The structure of CCP 11 from FH 10–11 overlaid well with the structure of CCP 11 from the FH 11–12 structure; moreover, CCP 12 solved in the current work overlaid well on CCP 12 from FH 12–13 (Supplementary Fig. S3 and Table S1). That module structures were conserved in FH 10–11, FH 11–12 and FH 12–13 endorsed construction of models of longer FH segments by concatenating bi-module structures (see below). While CCP 10's structure does not resemble the structures of other FH CCPs (backbone rmsd > 2 Å; Table S1), it is similar to MCP CCP 3^53^ (Supplementary Fig. S4a). CCP 12 is very similar in structure to CCP 2 of complement receptor type 2^54^ (Supplementary Fig. S4b). FH CCPs 11 and 12 resemble one another and are similar to FH CCPs 18 and 19^36^ (Supplementary Fig. S4c and Table S1).

### NOE-derived structures are not under-restrained or over-restrained

Within each bi-module, ~ 120 NOEs were identified between modules and their linker (Table 2). Eight NOEs were found directly between CCPs 10 and 11, compared to 38 NOEs between CCPs 11 and 12; thus, a network of NOEs that was more extensive for FH 11–12 than FH 10–11 defines the inferred inter-modular orientations. Indeed, the FH 10–11 structures exhibited good convergence when overlaid over both modules, and a comparable result was obtained for FH 11–12. Moreover, relaxation data (Supplementary Fig. S5) showed that inter‐modular linkers are not more mobile than other extended regions of the polypeptide. Nonetheless, several further steps were taken to check that these FH 10–11 and FH 11–12 solution structures were neither over-restrained nor under-restrained.

First, bi-module structures were recalculated using an ensemble-based simulated annealing protocol (in Xplor-NIH).[55], [56] This allows simultaneous generation of an ensemble of *n* conformers that, between them, satisfy all NOE-derived distance restraints. With the use of an ensemble size (*n*) of 2, ensemble members turned out to be nearly identical with one another and to the outcome of a standard simulated annealing protocol (i.e., effectively, *n* = 1); thus, we found no evidence that FH 10–11 or FH 11–12 adopts multiple conformers interconverting on the NOE timescale.

Second, NOE (only)-derived structures of FH 10–11 were fit to the FH 10–11 SAXS data (using CRYSOL^57^) with discrepancies (χ) (between data and model) of 1.3–1.6; equivalent values for FH 11–12 were 1.6–2.2 (Supplementary Fig. S6a). Thus, for both bi-modules, the NMR-derived structures provide a good fit to the scattering data. Furthermore, in an *ab initio* approach, the average structures of both bi-modules were reconstructed from SAXS data (using DAMMIF^58^). Within each set of 10 independent reconstructions, highly similar shapes were obtained, with low (< 1.0) normalized spatial discrepancies.^59^ Importantly, discrepancies between these models and high-resolution NMR-derived structures were small (χ = 0.9–1.2) in both cases. When averaged and volume-fitted *ab initio* shapes were superimposed with the corresponding NMR ensembles using SUPCOMB,^59^ good spatial agreement was observed (Supplementary Fig. S6b), thus validating the NMR-derived structures.

Finally, structure calculations (with ensemble sizes of 1 and 2) were conducted for each bi-module with incorporation of both NOE and SAXS data^60^ as experimental structural restraints.^61^ These calculations yielded structures (Table S2) that were nearly identical with NOE-only structures and exhibited no NOE violations > 0.5 Å.

### CCPs 11 and 12 are more intimately associated than CCPs 10 and 11

**Fig. 5:**
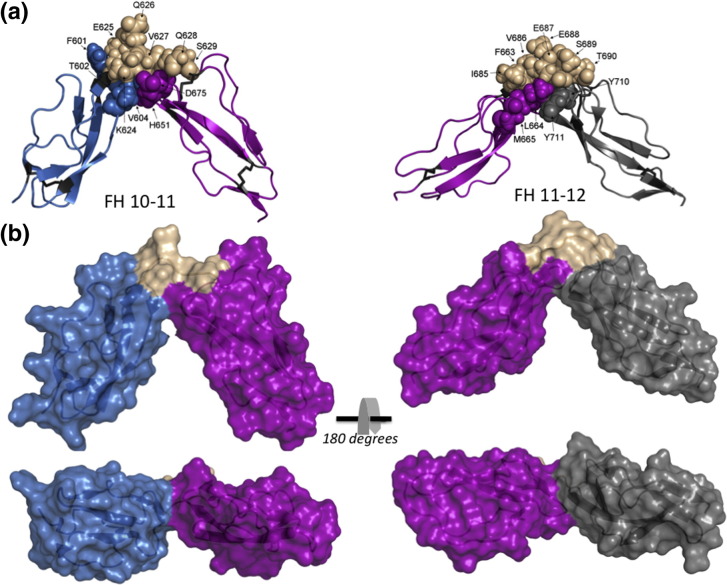
Inter‐modular interfaces in FH 10–11 and FH 11–12. (a) Side chains of residues involved in the interfaces drawn as color-coded labeled spheres: blue for CCP 10, magenta for CCP 11 and gray for CCP 12; wheat for the CCP 10–11 or the CCP 11–12 linker. (b) With the use of identical color coding, solvent-accessible surfaces are drawn, illustrating more extensive contacts between CCPs 11 and 12 despite larger tilt angle between CCPs 10 and 11.

Bi-modules FH 10–11 and FH 11–12 are bent (or tilted) by ~ 118° and ~ 90°, respectively, away from a linearly extended form. The surface area effectively buried between the N-terminal (CCP 10) and the C-terminal (CCP 11) halves of FH 10–11 (using the peptide bond between the middle two residues of the linker as a boundary) is only ~ 360 Å^2^, consistent with detection of just eight NOEs between these modules. An inferred salt bridge (Supplementary Fig. S7) links the first residue of the linker (Lys624, the last residue of CCP 10 β-strand H) to Asp675 of the CCP 11 FG β-turn. Residues Thr602 and Val604 from the CCP 10 β-strand E (paired with CCP 10 β-strand H) participate in inter‐modular van der Waals contacts with CCP 11 residues His651 and Asp675 (in BD loop and FG β-turn, respectively). All four contribute, along with alkyl portions of linker residues Lys624, Val627 and Gln628, to a hydrophobic pocket wedged between the modules (Fig. 5a). Polar side chains of linker residues Glu625, Gln626 and Ser629 are solvent exposed on the convex surface of the inter-modular bend.

In FH 11–12, side chains of the DE loop and β-strand E of CCP 11 (Pro661, Arg662, Phe663 and Leu664) make extensive van der Waals interactions with alkyl portions of linker residues (while polar portions are displayed on the convex surface of the bend) and with a segment of the BD loop of CCP 12 (Tyr710, Tyr711 and Gly712) (Fig. 5b). All these side chains are substantially buried in a hydrophobic pocket significantly bigger than the equivalent pocket in FH 10–11—the surface area buried between modules 11 and 12 is ~ 640 Å^2^.

In summary, CCPs 11 and 12 are welded together at an ~ 90° tilt in the structure calculation by a substantial number of mutually compatible NOEs reflecting extensive interactions and burial of a sizeable surface area. Modules 10 and 11 are mutually orientated at an even greater bend angle to accommodate (NOE-supported) interactions between strand E of CCP 10 and the CD and FG loops of CCP 11. Only a small surface area is buried between CCPs 10 and 11, but a Lys624–Asp675 salt bridge may stabilize it.

### FH 10–12 can be modeled from SAXS data and concatenation of bi-modules

Samples of FH 10–12 did not yield NMR spectra suitable for structure determination. This problem, encountered in previous efforts to solve triple-CCP‐module structures,[33], [52] was ascribed to anisotropic tumbling yielding lower *T*_2_ values than a globular protein of comparable mass. Broad peaks and crowded spectra precluded identification of sufficient inter-modular and module-to-linker NOEs without more elaborate isotopic labeling. Nonetheless, FH 10–12 yielded useful SAXS data. Three methods were used to combine results of SAXS for FH 10–12 and NMR for FH 10–11 and FH 11–12.

**Fig. 6:**
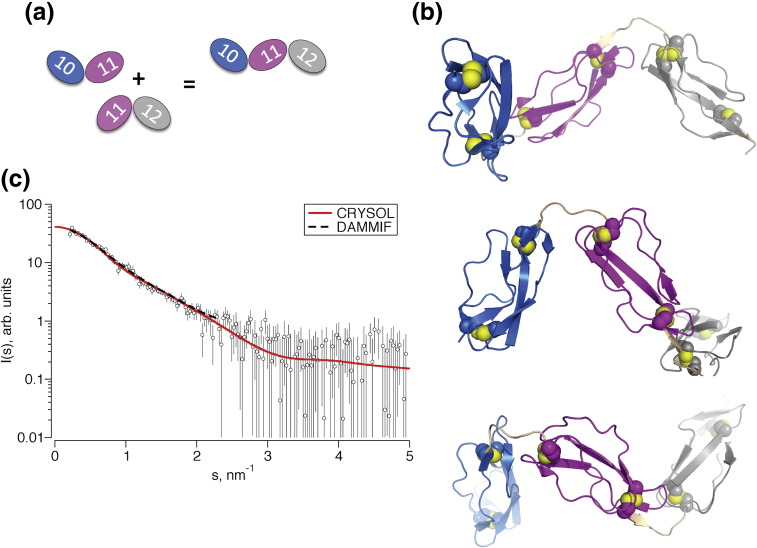
Structure of FH 10–12. (a) Overview of construction of the FH 10–12 model from concatenation of the FH 10–11 and FH 11–12 NMR structures. (b) Cartoon representation of a model of FH 10–12 built by concatenating structures of FH 10–11 and FH 11–12. Models are rotated about the horizontal axis by 90° and 180° relative to the topmost representation, respectively (for color coding, see Fig. 4). (c) Scattering curve of FH 10–12 fitted to the concatenated structure allows validation of the model.

First, a model of FH 10–12 built by concatenating FH 10–11 and FH 11–12 structures (Fig. 6a and b) was validated on the basis of successful fitting to SAXS data for FH 10–12 (χ = 0.8) (Fig. 6c) using the same method (see above) as employed for bi-modules. Second, in an *ab initio* approach, also similar to that described for the bi-modules, the average solution structure of FH 10–12 was reconstructed from SAXS data. As with the bi-modules, independent reconstructions yielded similar shapes (average normalized spatial discrepancies = 0.73 ± 0.12), while discrepancies between models and experimental data were low (χ = 0.88 ± 0.01), whereas the total excluded dummy-atom volume of the model matched the expected monomeric molecular mass. Encouragingly, the average *ab initio* shape superimposed well with, and thereby validated, the concatenated NMR model of FH 10–12 (Supplementary Fig. S2b). Finally, a combined FH 10–12 NOE list was compiled by concatenating FH 10–11 and FH 11–12 NOEs in a way that eliminated duplication or conflict of NOEs originating from the mutual CCP 11. Then, FH 10–12 structures were calculated using simulated annealing, employing as restraints both SAXS data and the combined NOE list. As with bi-modules, ensembles (*n* = 1 or 2) of structures were derived, with energy terms calculated as ensemble averages. Calculated FH 10–12 structures were in good agreement with the concatenated model, with each other and with both NOE and SAXS data.

**Fig. 7:**
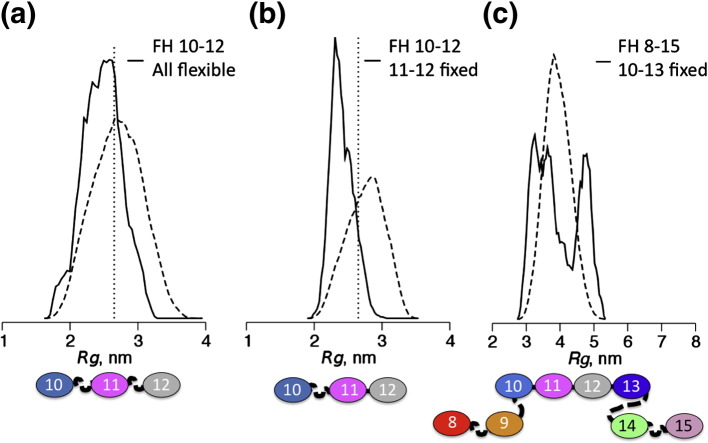
Applications of the ensemble optimization method. (a) In FH 10–12, individual CCPs were defined as rigid bodies but linkers were allowed to be flexible. Comparison of size distributions shows that an ensemble of structures selected from the pool (continuous line) that fit optimally to the SAXS data for FH 10–12 is more compact than the pool of random structures (broken line). (b) As in (a) except that, in this case, only the CCP 10–11 linker was permitted flexibility. Size distributions are consistent with a rigid arrangement of CCPs 10 and 11 despite limited contacts between modules. (c) An application of EOM to FH 8–15; all individual CCPs along with CCP pairs 10–11, 11–12 and 12–13 were defined as rigid bodies while other linkers were treated as flexible. The selected ensemble indicates a mixture of compact and extended conformers consistent with flexible attachment of CCPs 8 and 9 to a compact, relatively rigid core of CCPs 10–15.

The ensemble optimization method (EOM)^62^ was used to investigate flexibility of FH 10–12. In an initial application, NMR-derived structures of individual CCPs 10, 11 and 12 were used as rigid bodies and linker regions defined as flexible chains of dummy atoms to generate a pool of random conformations. Sets of conformers from the pool whose combined theoretical scattering pattern best fit the experimental data were then selected and compared to the whole pool of conformers. The size distribution of the selected ensemble (Fig. 7a) was narrower than that of the random pool and skewed toward compact conformations. Thus, the results of the EOM analysis are consistent with rigidity of modular connections and FH 10–12 not being significantly flexible or particularly extended. A subsequent EOM iteration was based on the observation of significantly fewer NOEs between CCPs 10 and 11 than between CCPs 11 and 12. Thus, CCPs 11 and 12 along with their linker were defined as a rigid body, while the 10–11 linker was treated as flexible. The resulting narrow size distribution (Fig. 7b) of the selected ensemble suggests that the CCP 10–11 linker is not significantly flexible in the FH 10–12 context, notwithstanding the relatively small surface area buried between modules.

### Structure of FH 10–13 and reinterpreting SAXS data for FH 10–15 and FH 8–15

**Fig. 8:**
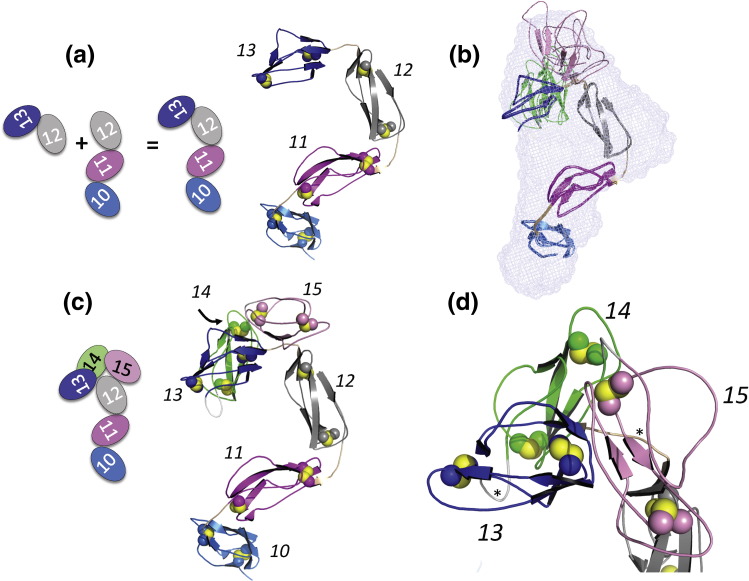
A model of FH 10–13 helps interpret SAXS data for FH 10–15. (a) Cartoon representation of a concatenated model of FH 10–13. (b) Two typical members of a convergent set of molecular structures that fit well to SAXS data for FH 10–15 interpreted on the assumption that CCPs 10–13 retain the spatial arrangement shown in (a), shown overlaid with the reconstructed SAXS envelope from DAMMIF. (c) A cartoon representation of CCPs 10–15 derived from SAXS data. (d) Zoom-in on CCPs 13–15 of SAXS-derived model; CCPs 14 and 15 bend over the CCP 12–13 linker in this model. Asterisks indicate 13–14 and 14–15 linkers that were built into the fitted structure manually (using MODELLER) since they were treated as dummy atoms during the fitting procedure.

FH 10–11, FH 11–12 and FH 12–13^35^ are all relatively rigid, and FH 10–12 lacks significant flexibility. Thus, inter‐modular angles are likely to be preserved between bi-module and tetra-module contexts. Hence, a concatenated model of FH 10–13—based on the three bi-module structures—should be reliable. MODELLER was used to construct such a model (Fig. 8), which shows how the curvature of FH 10–11 does not follow the arc described by FH 11–13, resulting in an out-of-plane zigzag structure. This model allowed reinterpretation of previously analyzed SAXS data for FH 10–15 and FH 8–15 with a less ambiguous outcome. Given that sequence directionality can be reliably inferred on the basis of the concatenated model of FH 10–13, rigid-body refinement of FH 10–15 consistently yields conformations with a compact C-terminal structure (Fig. 8); in these conformations, CCPs 14 and 15 interact intimately with CCP 13, which could have functional implications for the regulation of complement by FH as discussed below.

To assess the effect of adding CCPs 8 and 9 to FH 10–15, we conducted EOM analysis on FH 8–15. Lacking information to the contrary, all linkers excluding CCPs 10–11, 11–12 and 12–13 were defined as flexible. It was found that significant populations of both extended and compact inter-modular linker conformations were required in order to fit the data, resulting in a broad and bi-modal population distribution (Fig. 7c). Assuming that the FH 10–15 core remains rigid in the context of FH 8–15, this observation implies that CCPs 8 and 9 are not tightly associated with the other six modules. Interestingly, both SAXS data (Fig. 3) and a [^1^H,^15^N]HSQC spectrum collected on recombinant FH 8–9 (data not shown) indicated that this bi-module is as well folded as CCP 11–12 or 12–13. In conclusion, structurally autonomous CCPs 8 and 9 (connected to each other and to CCP 10 with well‐conserved linkers) adopt a much less intimate association with CCPs 10–13 than do CCPs 14 and 15; this observation supports the idea that the variable 13–14 linker and structural dependency of CCP 14 on its neighbors impose a uniquely compact arrangement of CCPs 14 and 15 with CCPs 10–13.

## Discussion

Like all known CCP structures^63^ (except CCP 13^35^), CCPs 10, 11 and 12 approximate to flattened prolate spheroids in which N and C termini occupy opposite poles. Given relatively small cross-sectional areas at intersections with neighbors, there are limited ways in which a long string of CCPs (such as FH) could attain the level of rigidity presumably needed for functional cooperation between remote binding sites. The two N-terminal modules of complement receptor type 2, for example, exhibit side-by-side interactions because the eight-residue inter-modular linker is long enough to allow a 142° inter‐modular bend (away from linearity).^54^ The rod-like architecture of FH CCP 19–20, on the other hand, arises from its minimal three-residue linker that restricts tilt and ensures interactions between loops on neighboring modules so as to also limit twist.[30], [31]

In the current work on FH 10–11, FH 11–12 and FH 10–12, we describe two CCP–CCP junctions that encompass six-residue inter-modular linkers. By working in solution, we minimized the risk for observation of potentially anomalous inter-modular tilts such as that observed between CCPs 18 and 19 in the crystal structure of FH 18–20.^36^ By combining NMR and SAXS, we circumvented the need for measurements of residual dipolar couplings wherein interaction with alignment media carries a theoretical risk of perturbing inter‐modular angles or dynamics. The relatively long linkers among CCPs 10, 11 and 12 facilitate large tilts and consequently significant inter-module interactions; their side chains also contribute to the hydrophobic “glue” between adjacent modules. A broadly similar arrangement of modules occurs in FH 12–13.^35^ Although previous SAXS-based studies of FH 1–5, FH 6–8 and FH 16–20 also indicated bent-back structures,^26^ high-resolution studies showed FH CCPs 1–3,[33], [34] FH 6–8^32^ and CCPs 19–20[36], [47], [64] to be linear or gently curved. Thus, overlapping bi-modules FH 10–11, FH 11–12 and FH 12–13, connected by linkers of six or eight residues and with tilts of about 80–100°, represent the most strongly tilted succession of four CCPs described to date. The FH conformation is ionic strength dependent and pH sensitive^65^; it is thus worth noting that equivalent His side chains (His651 in FH 11 and His773 in FH 13) feature prominently in both FH 10–11 and FH 12–13 interfaces while a salt bridge, Lys624–Asp675, may stabilize the small FH 10–11 junction.

That all three bi-modules exhibit only limited inter‐modular flexibility is consistent with the notion that the middle portion of the FH molecule needs flexional reinforcement if the ends (that carry the binding sites) are to be held in (one or more) preferred juxtaposition(s). An assumption that the inter-modular angles observed in the bi-modules 10–11 and 11–12 would persist in the tri-module FH 10–12 was validated by SAXS analysis. Therefore, it was reasonable to concatenate the three bi-modules to reconstruct CCPs 10–13, thus allowing us to trace the path in space of four modules within the central portion of FH. In agreement with a previous analysis of SAXS data for FH 10–15, CCPs 10–13 in this model do not all bend in the same direction to form a horseshoe-like shape. Instead, they form an out-of-plane S-shape or zigzag because the CCP 10–11 bend does not follow the curvature of CCPs 11–13.

We could not extend this instructive NMR-supported exercise to CCPs 14 and 15, as it was impossible to prepare the requisite samples of FH 13–14 or FH 14–15. No suitably stable samples were produced despite repeated attempts to exploit approaches that had previously proved successful for numerous CCP module pairs and longer constructs. This may be a host-specific effect; for example, low stability may arise from the lack of appropriate N-glycosylation^66^ or the presence of a site in folded CCP 14 that is uniquely susceptible to a *Pichia pastoris* protease despite addition of protease inhibitors. Nonetheless, several strands of evidence—described below—suggest that this finding in fact reflects a structural dependency of the common module, that is, CCP 14, on one or more immediate neighbors. Stabilizing interactions between CCP modules were noted in several previous studies,[67], [68] and there are two reports of strand swapping between neighboring CCP modules[69], [70]; moreover, a non-compactly folded CCP module was discovered in the GABA_B_ receptor.^71^ Thus, instability of CCP 14 in the absence of neighboring modules would not be unprecedented.

In previous SAXS-based studies of FH 10–15, one of two molecular models that fitted the experimental data featured CCP 14 folded back over CCP 13 with extensive, potentially mutually stabilizing, interactions between modules.^35^ The new solution structures of FH 10–11 and FH 11–12 allow a clearer interpretation of the FH 10–15 SAXS data; importantly, they reinforce the case for a near-180° bend between CCPs 13 and 14. Indeed, in the reinterpreted SAXS and NMR-based model of FH 10–15, CCP 14 contacts not only CCP 13 but also the 12–13 linker (Fig. 8). In the model, CCP 15 also contacts both the 12–13 linker and CCP 13. Notably, recombinant versions of single-module FH 13 and FH 15, but not FH 14, were readily prepared, yielding excellent NMR spectra. Moreover, relative well-behaved samples of FH 11–14, FH 10–15,^35^ FH 15–18 and FH 15–19^47^ also proved relatively straightforward to prepare. We were additionally able to prepare unlabeled NMR samples of the tri-module FH 13–15 although the quality of the ^1^H NMR spectrum was inferior to that of other tri-modules.^35^

Taking all these observations together implies that, for full stability, CCP 14 requires a stabilizing interface with CCPs 12, 13 and 15. On the other hand, CCPs 13 and 15 do not require CCP 14 (or CCP 12) for stability; rather, based on the lack of appropriate cross-peaks in the HSQC spectrum of FH 13–14, the attachment of putatively disordered CCP 14 to CCP 13, in the module-pair context, actually destabilizes CCP 13. Its highly conserved sequence is not suggestive of CCP 14 being unusual. Indeed, metaPrDOS^72^ does not predict a disordered structure for the CCP 14 sequence. On the other hand, its interface with CCP 13 must indeed be irregular. First, the 13–14 linker is the most variable (between orthologues) of any region within the whole of FH; for example, the CCP 13–14 linker is TSKTS in mice and NEEAKIQL in cows. All other 18 linkers are highly conserved. It is also the only linker of human FH that forms part of an occupied N-glycosylation sequence. Second, CCP 13 is poorly conserved in sequence across orthologues. Third, CCP 13 is less elongated than other CCPs and—importantly—lacks the DE loop and β-strand H that normally help to form a stable end-to-end interface with a subsequent CCP. By contrast, CCPs 8 and 9 that are structurally independent, have (or are predicted to have in the case of CCP 9^63^) classical CCP structures and are connected to each other and CCP 10 by conserved linkers are flexibly associated with the FH 10–13 unit.

Despite the low resolution of the NMR and SAXS-derived model of CCPs 10–15, it is highly plausible. According to the model, CCPs 12, 13, 14 and 15 form a compact structural unit unlike any other region of FH that has been described to date. CCPs 10, 11, 12 and 13 follow a more open zigzag path with 90° tilts (see Fig. 8). CCPs 12 and 13 act as a bridge between these two structural regions of FH 10–15 by participating in both of them. The linker between CCPs 13 and 14 is very probably unique in the FH molecule in that it has no structural role but forms a flexible and exposed loop on its surface. The positioning of CCP 13 in this model and the fact that CCP 14 is tilted away, leaving the C-terminal end of CCP 13 free, expose not only the non-conserved 13–14 linker but also an extensive highly variable region of CCP 13 encompassing β-strand B and the long BC loop (that includes a helical insertion), the variable DE loop and the aforementioned (non-conserved) N-glycosylation sequon adjacent to the C-terminal cysteine. Note that the presence within the central region of FH of large tilt angles or kinks is consistent, both with best-fit models from SAXS studies^25^ and with earlier transmission electron micrographs of full-length FH.^24^

While the sequential series of inter‐modular tilts between CCPs 10 and 13 contribute to flexional reinforcement, the purpose of an extreme tilt at 13–14 is less clear. Further studies would be needed to investigate whether this module pair could correspond to the “hinge” in FH that has been suggested^35^ as a means whereby the molecule can switch rapidly between two conformations—a compact or closed one (as reflected in FH 10–15) that holds the two binding sites in such a way that they have relatively low affinity for C3b^73^ and an “open” one in which the two ends are able to bind simultaneously, and with high affinity, to nonoverlapping sites on a single molecule of C3b (or adjacent C3b molecules) in the context of a self-surface and its polyanionic markers. More investigation is required to test whether flexional stress or exposure to the electronegative environment found at the host surface disrupts the 13–14 interface. Under these circumstances, module 14 would be likely to become intrinsically disordered, creating a very flexible segment within the FH molecule and allowing terminal regions to cooperate in binding to a target.

## Materials and Methods

### Protein production

Using procedures similar to those previously described,[30], [74] we produced recombinant proteins in *P. pastoris*. Very briefly, DNA encoding FH 10–11 (amino acid residues 566–687), FH 11–12 (amino acid residues 627–747), FH 10–12 (amino acid residues 566–745), FH 13–14 (amino acid residues 748–865) or FH 14–15 (amino acid residues 808–929) (numbers refer to unprocessed initial human gene product) was PCR amplified from FH cDNA. Each insert was ligated into a TOPO plasmid vector subsequently used to transform Top10 *Escherichia coli* cells (Invitrogen). The amplified plasmid was digested (PstI and XbaI), and target DNA was ligated into the pPICZα B vector (Invitrogen), 3′ of DNA coding for the *Saccharomyces cerevisiae* α-mating factor secretion signal that directs protein production into the secretory pathway. Following amplification in *E. coli* cells and SacI linearization, we used the plasmid to transform *P. pastoris* KM71H. Transformed yeast cells were fermented, and gene expression was induced with methanol. Following centrifugal cell removal, we undertook purification from media using ion-exchange chromatography, typically on SP-Sepharose (GE Healthcare), normally followed by size-exclusion chromatography on a Hi-Load 16/60 Superdex 75 120-mL column (GE Healthcare). Proteins bearing high-mannose N-linked glycans were treated (after first purification step) with Endo H_f_ (New England Biolabs). Purification was monitored by sodium dodecyl sulfate–polyacrylamide gel electrophoresis under reducing and nonreducing conditions; the molecular weight of the purified material was validated with Fourier transform–ion cyclotron mass spectrometry. For ^15^N enrichment, cells were grown in minimal media supplemented with ^15^N-labeled ammonium sulfate (Isotec, Sigma-Aldrich), glycerol, basal salts, trace elements and vitamins and were subsequently induced with methanol. For ^13^C,^15^N enrichment, a similar protocol was used but cells were grown in media containing ^13^C glucose (instead of glycerol), and prior to induction with ^13^C methanol, ^13^C glycerol (Isotec, Sigma-Aldrich) was added to facilitate derepression of the alcohol oxidase promoter.

### Collection and processing of NMR data and assignment of spectra

NMR spectra were collected at 298 K on samples containing 10% (v/v) D_2_O in 5-mm NMR tubes, on Bruker AMX 800‐ and 600‐MHz instruments fitted with cryoprobes. Gradual proteolysis occurred in the case of FH 10–11, necessitating production of three ^13^C,^15^N-labeled NMR samples (at 400, 150 and 500 μM in 20 mM potassium phosphate buffer, pH 6.7). For FH 11–12, one 650‐μM ^13^C,^15^N-labeled sample (in 20 mM potassium phosphate buffer, pH 6.3) sufficed. In the case of FH 13–14, production of a properly folded and stable sample suitable for NMR proved difficult (see Results) and only a ^15^N-labeled sample (150 μM in 20 mM potassium phosphate buffer, pH 6.6) was prepared. Topspin version 1.3 (Bruker) was used to set up the experiments, to acquire data and for initial data processing. The data were brought to their final format by processing using AZARA (W. Boucher, Department of Biochemistry, University of Cambridge, UK).

Resonance assignments were accomplished using the CCPN Analysis software.^75^ For backbone assignments, HBHANH (for FH 11–12), HBHA(CO)NH, CBCANH, CBCA(CO)NH, HNCO and HN(CA)CO experiments were collected. For side-chain assignments, HCCH–total correlated spectroscopy (TOCSY), H(C)(CO)NH–TOCSY and (H)C(CO)NH–TOCSY experiments were acquired along with (two-dimensional) (HB)CB(CGCD)HD and (HB)CB(CGCDCE)HE experiments for assigning aromatic side chains.

### Solution structure determination

Including the nonnative N-terminal sequence (EAAG and EAEAAG in FH 10–11 and FH 11–12, respectively), 88% of triple-resonance assignments, including 97% of backbone atoms for FH 10–11 and 88% of triple-resonance assignments, including 95% of backbone atoms for FH 11–12, were completed. Of the theoretically assignable backbone atoms, the following were missing: Pro632 (CO), Pro633 (CO) and Asp675 (N and HN) in the case of FH 10–11 and Glu621 (H, HN, CO, C^α^), Ala622 (H, HN and C^α^), Pro632 (CO, C^α^), Pro633 (CO), Asp675 (N, HN), Pro707 (CO) and Ile747 (CO) in the case of FH 11–12.

In one out of twelve in the case of FH 10–11 (Pro618) and one out of ten in the case of FH 11–12 (Pro708), X-Pro linkages were defined as *cis*, while the remainder were defined as *trans* as judged by chemical shift differences between Pro ^13^C^β^ and ^13^C^γ^ atoms,^76^ by strong NOEs observed between H^α^ (Xaa^*i* − 1^) and H^α^ (Pro^*i*^) and between H^α^ (Xaa^*i* − 1^) and HN (Xaa^*i* + 1^) and by no or weak detectable NOEs between H^α^ (Xaa^*i* − 1^) and H^δ^ (Pro^*i*^). Based on mass spectrometry, all eight cysteine residues in both FH 10–11 and FH 11–12 were inferred to be in the oxidized state. Specific disulfide bridges were incorporated into the final rounds of structure calculations on the basis of NOEs (reinforced by a precedent established by previous studies of CCPs). Approximately 3% (for FH 10–11) and 14% (for FH 11–12) of peaks in the ^15^N-edited NOE spectroscopy spectra were assigned manually while, for both constructs, ~ 8% of peaks in the ^13^C-edited NOE spectroscopy spectra were also assigned manually.

Remaining NOEs were assigned by a combination of automated assignment and structure calculation. Thus, seven cycles of CYANA 2.1^49^ were employed for each of FH 10–11 and FH 11–12 to generate lists of NOE-derived distance restraints and sets of preliminary structures. Once CYANA-generated parameters met published quality criteria,^49^ distance restraints were transferred into the program CNS,^50^ using the CCPN Format Converter program. Working within CNS allows structure refinement against explicit water.

### Assessing and comparing structures

The quality of structures was assessed using PROCHECK^77^ (see Table 1). A hydrogen–deuterium exchange experiment was performed to cross-validate hydrogen bonds observed in the NOE-derived structures; for this purpose, lyophilized protein was transferred to deuterated buffer, whereupon slowly exchanging amide protons were identified in a [^15^N,^1^H]HSQC spectrum collected 15 min after exposure to D_2_O. Secondary structure elements were identified using STRIDE.^51^

The Web server VADAR 1.8^78^ was used to establish the solvent accessible surface area (Å^2^); the buried surface area was computed as (SA module_*i*_ + SA module_*j*_) − SA bi-module_*ij*_, where CCP *i* was considered to encompass one residue before its Cys(I) and three residues after its Cys(IV), and CCP (*j*) boundaries were considered the third residue before its Cys(I) and one residue after its Cys(IV).

Inter‐modular tilt, twist and skew angles[29], [79] were determined using an in-house script†; a vector was chosen between the principal axis of the inertia tensor (the *z*-axis) and the C^α^ of Leu616 (CCP 10), Trp678 (CCP 11) or Trp738 (CCP 12), respectively, and with module boundaries defined as Cys(I), that is, Cys569 (CCP 10), Cys630 (CCP 11) or Cys691 (CCP 12), and Cys(IV), that is, Cys623 (CCP 10), Cys684 (CCP 11) or Cys744 (CCP 12), respectively.

Combinatorial extension^80^ was employed to compare each experimentally determined CCP structure within the complement system against the closest-to-mean individual structures determined herein.

### SAXS data collection and interpretation

Synchrotron radiation X-ray scattering data were collected on the X33 beamline of the European Molecular Biology Laboratory (Deutsches Elektronen‐Synchrotron, Hamburg) using a 1M PILATUS pixel detector (Dectris, Switzerland) and eight frames of 15-s exposure time. Samples were analyzed at 20 °C, using protein concentrations of 0.3–10.7 mg/mL in phosphate‐buffered saline. The sample-to-detector distance was 2.7 m, covering a range of momentum transfer 0.08 ≤ *s* ≥ 6.0 nm^− 1^ [where *s* = 4πsin(θ)/λ , with 2θ being the scattering angle and λ = 0.15 nm being the X-ray wavelength]. Based on comparison of successive 15-s frames, samples in which radiation damage was detected were further scrutinized and only frames showing no significant change in intensity were used for subsequent data analysis.

Data from the detectors were normalized to the transmitted beam intensity and averaged, and the scattering of buffer solutions was subtracted. The difference curves were scaled for solute concentration. Data manipulations were performed using the ATSAS software package[81], [82] utilizing the recently developed DATTOOL library for averaging, merging and extrapolation to infinite dilution from concentration series (Guinier regions for samples at several concentrations, showing linearity; Supplementary Fig. S8). The forward scattering *I*(0) and radius of gyration *R*_g_ were determined from Guinier analysis,^83^ assuming that, at very small angles (*s* ≤ 1.3/*R*_g_), the intensity is represented as *I*(*s*) = *I*(0)exp((*sR*_g_)2/3). These parameters were also estimated from the full scattering curves using the indirect Fourier transform method implemented in the program GNOM,^84^ along with the distance distribution function *p*(*r*) and the maximum particle dimensions *D*_max_. Molecular masses of solutes were estimated from SAXS data by comparing the extrapolated forward scattering with that of a reference solution of bovine serum albumin (66 kDa).

### Fit of the NMR structures of FH fragments to the SAXS data

The fits of the NMR-derived structures of FH fragments to the SAXS data were conducted using the program CRYSOL.^57^ CRYSOL calculates the partial scattering amplitudes of proteins from their atomic coordinates, taking into account the hydration layer and excluded solvent volume. The number of harmonics was set to 50 and 256 points computed across the entire data range (0 < *s* < 5 nm^− 1^). The NMR-derived structures were also fitted to the SAXS data with Xplor-NIH using 500 solid angles and 100 data points evenly distributed in a cubic spline constructed using the SAXS data within the range specified above.^85^

### Analysis of flexibility

Analysis of inter-module flexibility and size distribution of possible conformers consistent with the measured scattering data was conducted using the EOM.^62^ The EOM selects an ensemble of possible conformations of input rigid bodies (using the CCP modules 10, 11 and 12 determined in this study and the remaining modules as described previously^34^) and user‐defined regions of flexible sequence (here defined as the linkers between CCP modules) from a pool of 10,000 randomly generated models, using CRYSOL to calculate the theoretical scattering profiles and a genetic algorithm, GAJOE, to select the representative set that best describes the experimental data.

### *Ab initio* shape determination and molecular modeling

Low-resolution‐shape envelopes were determined using the *ab initio* bead-modeling program DAMMIF.^58^ DAMMIF represents the particle as a collection of *M* densely packed beads inside an adaptable and loosely constrained search volume compatible with the experimentally determined *R*_g_. Each bead is randomly assigned to solvent (index = 0) or solute (index = 1), and the particle structure in solution is described by a binary string of length *M*. Disconnected strings of beads are rejected, and the scattering amplitudes are calculated. Simulated annealing is then used to search for a compact model that minimizes the discrepancy between the experimental and calculated intensities of momentum transfer. The results of 10 independent DAMMIF reconstructions were compared using SUPCOMB13^59^ to determine the most representative (typical) model. Averaged DAMMIF models were also determined using DAMAVER, and these models were adjusted such that they agree with the experimentally determined excluded volume using DAMFILT.^86^

Molecular modeling used, as rigid bodies and where appropriate, the same structures as used for EOM. Rigid-body models were generated using the program CORAL, an advanced version of the rigid-body modeling program BUNCH[81], [87] where linkers/loops between individual subunits are represented as random polypeptide chains. The results of 10 independent CORAL runs were analyzed using the programs SUPCOMB13 and DAMAVER to identify the most representative/typical models.

### Combining SAXS and NOEs

A concatenated FH 10–12 NOE list was created by merging experimentally derived FH 10–11 and FH 11–12 NOE lists. Only NOEs from the FH 10–11 list that involve residues in (and between) CCP 10, the CCP 10–11 linker and the N-terminal half of the three‐dimensional structure of CCP 11 were used; likewise, the only NOEs extracted for use from the FH 11–12 list were those in (and between) the C-terminal half of the three‐dimensional structure of CCP 11, the CCP 11–12 linker and CCP 12. This procedure, similar to that described previously,^56^ was designed to remove potential duplications and conflict involving the common CCP 11.

Structure calculations, incorporating the SAXS data for FH 10–12 and the concatenated NOE list, were carried out in Xplor-NIH version 2.30.^55^ A naive model of FH 10–12, prepared using MODELLER 9v10^88^ by overlaying closest-to-mean structures of FH 10–11 and FH 11–12 on the common CCP 11, was used as a starting structure. In the first stage of a simulated annealing protocol,^55^ the temperature was lowered from an initial 2000 K to 600 K in 50-K intervals, with 300 steps of molecular dynamics at each temperature increment; within this temperature interval, the energy terms were progressively introduced by multiplicative ramping. In the second stage, the structures were cooled from 600 K to 100 K, in increments of 25 K, with 300 steps of molecular dynamics at each temperature; potential terms were statically set to the top values used in the first stage of cooling. Two calculations were carried out: first, a set of 100 standard protein structures was calculated; second, 100 ensembles of structures (with number of members *N* = 2 in each ensemble) were calculated. In the ensemble calculation, the energy terms were calculated as an ensemble average, as described before.^61^ Similar calculations were performed for FH 10–11 and FH 11–12, using the relevant SAXS data and NOE lists and NOE-only derived structures as starting structures.

### Relaxation data

Measurements of backbone ^15^N *T*_1_ and *T*_2_ and ^1^H,^15^N NOEs were conducted using a 600-MHz magnet and 0.45‐nM and 0.65‐mM (for FH 10–11 and FH 11–12, respectively) samples in 20 mM potassium phosphate buffer, pH 6.7 (FH 10–11) or pH 6.3 (FH 11–12). Delays used for *T*_1_ (ms) were (FH 10–11) 51.2, 51.2, 301.2, 501.2, 601.2, 751.2, 851.2 and 901.2 or (FH 11–12) 51.2, 301.2, 501.2, 601.2, 701.2, 801.2 and 901.2. Delays used for *T*_2_ (ms) (FH 10–11) were 16.96, 16.96, 33.92, 67.84, 101.76, 118.72, 135.68 and 152.64 or (FH 11–12) 16.96, 33.92, 67.84, 84.8, 101.76, 118.82 and 135.68. For heteronuclear NOE measurements, a reference experiment with a 5-s relaxation delay was followed by a second spectrum recorded with ^1^H saturation achieved by a train of 120° pulses applied for the last 3 s of the 5-s delay to attain NOE build up. The NMR data were processed using AZARA, and spectra were assigned using Analysis.^75^ Relaxation rates were obtained by fitting a single-exponential decay to the extracted cross-peak height of each residue using nonlinear fitting. In the case of FH 10–11, the following native residues were excluded from the analyses: Glu566, Arg567, Lys583, His613, Ser652 and Gly676 (signals form the backbone amides were too weak); Asp581, Val589, Val592, Phe595, Cys597, Phe601, Val609, Val627, Thr645, Tyr649 and Ile671 (due to overlap); and Asp675 (amide not found). In the case of FH 11–12, the following native residues were excluded: Ser652, His735 and Gly736 (weak signals); Tyr658, Leu697, Gln703, Tyr709, Ile731 and Cys733 (due to overlap); and Asp675 (amide not found).

### Accession numbers

NMR data and structure coordinates have been deposited at the BMRB (*B*iological *M*agnetic *R*esonance *B*ank) and at the PDB (*P*rotein *D*ata *B*ank). Accession numbers are as follows:

FH 10–11: PDB, 4b2r; BMRB, 18604
FH 11–12: PDB, 4b2s; BMRB, 18599

## Glossary

Complement: Approximately 30 proteins involved in multiple aspects of innate and acquired immunity.

Alternative pathway: Complement activation via spontaneous C3 hydrolysis; in contrast, antibody–antigen complexes and polysaccharides are needed to activate the classical and lectin pathways, respectively.

C3: A complement protein that is cleaved by C3 convertase into C3a and C3b; cleavage activates a thioester group in C3b that is able to react with any nearby nucleophile.

C3(H_2_O): A form of C3 in which the thioester has been spontaneously hydrolyzed; resembles C3b in domain arrangements and ability to form part of a C3 convertase complex.

C3 convertase: Binary complex—C3(H_2_O).Bb or C3b.Bb—that cleaves C3 to C3a and C3b; formation involves C3b or C3(H_2_O) binding proenzyme factor B that subsequently gets cleaved (and activated) by factor D to Bb.

CCP modules: Domains, which predominate among regulators of complement activation, containing ~ 60 amino acid residues and two disulfides.

## Footnotes

† Available from www.bionmr.chem.ed.ac.uk/bionmr/public_html/ccp-db.html
