# Supplementary material for: Solution Structure of CCP Modules 10–12 Illuminates Functional Architecture of the Complement Regulator, Factor H
Source: J Mol Biol. 2012 Dec 14;424(5):295–312. doi: 10.1016/j.jmb.2012.09.013 (PMC4068365; doi:10.1016/j.jmb.2012.09.013)
Supplement: Supplementary file 1 — Supplementary materials [file mmc1.pdf]

Solution structure of CCP modules 10-12 illuminates functional architecture of the complement regulator, factor H

Elisavet Makou, Haydyn D. T. Mertens, Mateusz Maciejewski, Dinesh C. Soares, Ilias Matis, Christoph Q. Schmidt, Andrew P. Herbert, Dmitri I Svergun and Paul N. Barlow

Supplementary Data

Table S1 Comparison of new structures with existing CCP structures.

Table S2 Structural statistics of FH 10-12 model calculated according to a combined SAXS and NMR dataset (see Methods).

Figure S1 *Comparison of ^1H,^{15}N HSQC spectra for FH 13-14 (blue) and FH 13 (black)*. The spectrum of FH 13-14 (lower) shows little evidence of folded structure. Only a few cross peaks in the FH 13-14 spectrum correspond to folded FH 13, based on an overlay of the spectra (upper).

Figure S2 *Comparison of slowly exchanging amides in 10-11 and 11-12*. Slowly exchanging amides for FH 10-11 (upper) and FH 11-12 (lower). In each case, peaks in a ^1H,^{15}N HSQC spectrum that are detectable after a 15-minute exposure to D_2O are identified.

Figure S3 *Extrapolating to triple-module structures.* **(A)** CCP 11 of the FH 10-11 structure and CCP 11 from the FH 11-12 structure overlay well. **(B)** Overlays (orthogonal views) of the filtered average DAMMIF shape envelopes (mesh representation) with the concatenated FH10-12 structural model (data and CRYSOL fits shown in Fig. 6C in main text). **(C)** CCP 12 of the FH 11-12 structure and CCP 12 from the FH 12-13 structure also overlay well.

Figure S4 *Comparisons with the most similar CCP structures in the database* Sequence alignments and overlays of NMR-derived structures of FH 10, 11 and 12 with the CCP module structures to which they are most similar (see Table S1); details in text.

Figure S5 *Relaxation data for bi-modules.* **(A)** From the top, T_1, T_2 and heteronuclear NOE values, for FH 10-11, are plotted versus residue number. In each plot the mean, +/- standard deviation is indicated, calculated for residues with heteronuclear NOEs > 0.6. β-strands and the position of the linker are shown by the labeled, shaded boxes. Inverted triangles on the *x*-axis indicate Pro positions while star shapes denote residues that were excluded from the analysis due to overlap or low signal. (**B**) As in A, but for FH 11-12. The sequential occurrence of residues with unusual relaxation parameters may be interpreted in terms of motions on various timescales. For example the BD loop of CCP 11 (both in FH 10-11 and FH 11-12 is probably undergoing slow exchange as suggested by low T_2 values (and unremarkable T_1 values). The BD loop is less mobile (ns-timescale) in FH 10-11 (as evidenced by larger heteronuclear NOEs), presumably because it is stabilized by the interface with CCP 10. Likewise, the region just prior to strand D of CCP 12 is characterized by high heteronuclear NOEs, perhaps because it is participating in the interface with CCP 11. Notably, the two intermodular linkers are not markedly more mobile than other regions. Taking all this data together, there is little evidence of intermodular flexibility in these two bi-modules on the time-scales probed by NMR.

Figure S6 *SAXS was used to confirm NOE-derived structures of bimodules.* **(A)** SAXS data and CRYSOL fits of the mean NMR-derived structures to the SAXS data for FH 10-11 (left) and FH 11-12 (right) (**B**) Overlays (orthogonal views) of the filtered average DAMMIF shape envelopes (mesh representation) with the NMR ensembles of FH(10-11) (left), FH(11-12) (right).

Figure S7 *An inferred salt bridge at the interface between CCPs 10 and 11*. The side chains of participating residues are shown as sticks in the enlargement; cysteine side chains are shown as spheres. Color coding as in Figure 4C in main text.

Figure *S8* *Guinier plots for FH bimodules*. Guinier regions for FH10-11 (upper) and FH11-12 (lower) at several concentrations, showing linearity.

Table S1 – Comparison of new structures with existing CCP structures

**Protein; module; (PDB ID)**	**RMSD in Å (alignment length, gaps included)**			
	**FH 10**	**FH 11[a]**	**FH 11[b]**	**FH 12**
C1r; CCP 1; (1GPZ)	1.89 (59)	2.16 (58)	2.18 (58)	2.28 (58)
C1r; CCP 2; (1GPZ)	2.52 (34)	2.68 (34)	2.41 (35)	1.98 (38)
C1s; CCP 2; (1ELV)	2.20 (58)	2.31 (50)	2.45 (50)	2.05 (50)
C2; CCP1; (3ERB)	3.17 (51)	3.07 (50)	3.25 (50)	3.01 (50)
C2; CCP2; (3ERB)	2.13 (59)	2.45 (58)	2.68 (58)	2.88 (58)
C2; CCP3; (3ERB)	1.94 (57)	2.13 (56)	2.20 (56)	2.17 (56)
C4BPα; CCP 1; (2A55)	3.51 (59)	2.27 (58)	2.41 (58)	2.47 (58)
C4BPα; CCP 2; (2A55)	2.05 (58)	2.07 (52)	1.99 (52)	2.31 (57)
CR1; CCP 15; (1GKN)	2.81 (57)	2.26 (58)	2.24 (57)	2.37 (56)
CR1; CCP 16; (1GKN)	2.06 (58)	2.03 (51)	2.06 (51)	2.31 (57)
CR1; CCP 17; (1GKG)	2.80 (61)	2.14 (58)	1.99 (57)	2.09 (57)
CR2; CCP 1; (1LY2)	2.05 (59)	1.71 (58)	1.78 (58)	2.10 (58)
CR2; CCP 2; (1LY2)	1.94 (58)	1.70 (57)	1.82 (57)	1.41 (54)*
CRRY; CCP 1; (2XRB)	3.36 (58)	2.42 (57)	2.48 (58)	2.47 (56)
CRRY; CCP 2; (2XRB)	2.28 (58)	2.35 (53)	2.08 (53)	2.28 (57)
CRRY; CCP 3; (2XRB)	1.71 (59)	1.72 (59)	1.74 (59)	2.03 (58)
CRRY; CCP 4; (2XRB)	1.95 (57)	1.53 (54)	1.59 (54)	1.96 (56)
DAF; CCP 1; (1OK3)	2.39 (59)	2.20 (59)	1.89 (59)	2.25 (58)
DAF; CCP 2 (1OK3)	2.58 (56)	2.24 (57)	1.94 (56)	1.94 (58)
DAF; CCP 3; (1H03)	2.28 (58)	2.38 (57)	2.36 (57)	2.31 (57)
DAF; CCP4; (1H03)	1.75 (58)	1.59 (57)	1.62 (57)	1.79 (57)
FB; CCP 1; (2OK5)	2.94 (62)	2.61 (46)	3.86 (65)	3.10 (63)
FB; CCP 2; (2OK5)	2.01 (59)	2.21 (58)	2.43 (58)	2.72 (58)
FB; CCP 3; (2OK5)	2.04 (57)	2.16 (56)	2.24 (56)	2.47 (56)
FH; CCP 1; (2RLP)	2.73 (51)	2.04 (57)	2.09 (57)	2.39 (56)
FH; CCP 2; (2RLQ)	2.41 (57)	2.29 (54)	2.34 (55)	2.38 (56)
FH; CCP 3; (2RLQ)	1.97 (57)	2.08 (57)	2.14 (57)	2.47 (56)
FH; CCP 4; (2WII)	2.16 (57)	1.72 (57)	1.82 (57)	2.14 (55)
FH; CCP 5; [1]	2.24 (56)	2.42 (53)	2.45 (53)	2.37 (52)
FH; CCP 6; (2UWN)	3.16 (59)	2.51 (57)	2.19 (56)	2.53 (56)
FH; CCP 7; (2UWN)	2.89 (56)	3.05 (57)	2.85 (55)	3.56 (57)
FH; CCP 8; (2UWN)	2.00 (54)	1.80 (51)	1.95 (51)	2.25 (56)
FH; CCP 10; (this study)	-	2.04 (57)	2.15 (58)	2.37 (58)
FH; CCP 11[a]; (this study)	2.04 (57)	-	0.87 (59)	1.61 (58)
FH; CCP 11[b]; (this study)	2.15 (58)	0.87 (59)	-	1.57 (58)
FH; CCP 12; (this study)	2.37 (58)	1.61 (58)	1.57 (58)	-
FH; CCP 12; (2KMS)	2.19 (57)	1.21 (58)	1.26 (58)	1.25 (58)
FH; CCP 13; (2KMS)	2.83 (48)	3.28 (56)	3.61 (43)	3.40 (50)
FH; CCP 15; (1HFH)	2.53 (58)	1.90 (59)	1.89 (59)	1.84 (57)
FH; CCP 16; (1HFH)	2.89 (51)	1.79 (54)	1.89 (56)	1.81 (56)
FH; CCP 18; (3SW0)	1.96 (58)	1.25 (59)	1.38 (59)	1.80 (58)
FH; CCP 19; (2G7I)	1.93 (57)	1.18 (59)*	1.13 (58)*	1.70 (57)
FH; CCP 20; (2G7I)	2.08 (56)	2.10 (56)	2.49 (57)	1.69 (48)
MASP1; CCP 1; (3GOV)	1.92 (59)	2.11 (58)	2.17 (58)	2.42 (58)
MASP1; CCP 2; (3GOV)	2.43 (59)	2.50 (59)	2.42 (59)	1.97 (58)
MASP2; CCP 1; (1ZJK)	2.02 (59)	2.09 (58)	2.14 (58)	2.36 (58)
MASP2; CCP 2; (1ZJK)	2.25 (59)	2.24 (59)	2.26 (59)	1.98 (58)
MCP; CCP 1; (1CKL)	2.40 (55)	2.09 (57)	1.98 (57)	2.14 (57)
MCP; CCP 2; (1CKL)	2.60 (59)	2.88 (58)	3.00 (58)	2.79 (58)
MCP; CCP 3; (3O8E)	1.69 (59)*	1.77 (59)	1.77 (59)	2.27 (58)
MCP; CCP 4; (3O8E)	2.39 (59)	1.89 (55)	1.92 (55)	1.99 (54)
VCP; CCP 1; (1G40)	2.52 (58)	2.70 (59)	2.60 (59)	2.87 (58)
VCP; CCP 2; (1G40)	2.34 (57)	2.23 (51)	2.34 (51)	2.27 (50)
VCP; CCP 3; (1G40)	2.47 (58)	1.89 (58)	2.04 (58)	1.99 (57)
VCP; CCP 4; (1G40)	1.89 (56)	2.00 (56)	2.04 (56)	2.21 (55)

Supplementary Table S1. Shown are the results of pair-wise comparisons of FH 10, FH 11[a] (where [a] = from FH 10-11), FH 11[b] (where [b] = from FH 11-12) and FH 12 with all other individual CCPs of known structure within complement proteins based upon alpha-carbon (RMSD) values, using structural alignment program Combinatorial Extension (see Methods). For each CCP, inclusive module boundaries were one residue before Cys-I and the third residue after Cys-IV. Where structures were solved by both NMR and X-ray diffraction, the higher resolution X-ray structure was used for comparison. Where both liganded and unliganded structures were available, the highest resolution unliganded X-ray or NMR structure was used. Colour key used in table: Blue: 0 - 1.99 Å; Green: 2.00 – 2.99 Å; Red: 3.00 – 3.99 Å. Abbreviations used in Table: C4BPα = C4b-binding protein α-chain; CR = complement receptor; CRRY = rat Complement receptor 1-related protein Y; DAF = decay-accelerating factor; FB = factor B; FH = factor H; MASP1 / 2 = mannan-binding lectin-associated serine proteases 1 / 2; MCP = membrane cofactor protein; VCP = Vaccinia virus complement control protein. Some residues were not present (solved) in the electron density map for the C1r CCP 2 module crystal structure, and this explains the short structural alignment length (shown in brown). The best overlaying structures in each case are indicated by '*'.

[1]Coordinates downloaded from www.bionmr.chem.ed.ac.uk/bionmr/public_html/ccp-db.html .

Table S2 – Structural statistics for FH 10-12 ensemble *(n*= 2) calculations based on SAXS and NOE data

	FH10-11	FH11-12	FH10-12	
# representative ensembles	20	20	20	
Angle RMS (S.D)	0.62 (0.01)	0.57 (0.01)	0.65 (0.01)	
Bond RMS (S.D.)	0.004 (0.001)	0.004 (0.001)	0.005 (0.001)	
Improper RMS (S.D.)	0.38 (0.01)	0.33 (0.01)	0.45 (0.02)	
NOE RMS (S.D.)	0.02 (0.02)	0.02 (0.02)	0.03 (0.03)	
SAXS c^2	1.03 (0.04)	0.58 (0.57)	0.66 (0.02)	
**RMSD**				
**All Modules**				
All atoms	0.68 (0.05)	0.56 (0.05)	1.55 (0.27)	
Backbone atoms	0.40 (0.06)	0.33 (0.08)	1.41 (0.29)	
**Module 10**				
All atoms	0.60 (0.04)	-	0.71 (0.05)	
Backbone atoms	0.29 (0.03)	-	0.40 (0.06)	
**Module 11**				
All atoms	0.61 (0.04)	0.52 (0.04)	0.68 (0.07)	
Backbone atoms	0.28 (0.04)	0.23 (0.05)	0.34 (0.05)	
**Module 12**				
All atoms	-	0.47 (0.04)	0.67 (0.05)	
Backbone atoms	-	0.17 (0.03)	0.30 (0.04)	
**Intermodular angles {minimum-maximum [mean (S.D.)]}**				
Interface	CCP 10-11	CCP 11-12	CCP 10-11	CCP 11-12
Skew (°)	52-55 [54(1)]	84-89 [86(1)]	50-54 [52(1)]	76-95 [85(5)]
Twist (°)	93-108 [100(6)]	147-158 [154(3)]	93-108 [100(6)]	154-166 [160(3)]
Tilt (°)	101-106 [103(1)]	71-78 [74(2)]	102-119 [111(5)]	80-97 [89(6)]
**Intra-ensemble intermodular angle variability {minimum-maximum [mean (S.D.)]}[1]**				
Interface	CCP 10-11	CCP 11-12	CCP 10-11	CCP 11-12
Skew (°)	-4-2 [0(2)]	1-6 [3(2)]	-16-13 [-4(10)]	-7-7 [-2(3)]
Twist (°)	7-14 [11(2)]	-3-9 [3(4)]	7-14 [11(2)]	-3-9 [4(3)]
Tilt (°)	-2-2 [0(1)]	-3-1 [-1(1)]	-1-3 [1(1)]	2-11 [7(2)]
**Ramachandran assessment[2]**				
Most favoured (%)	52.9	56.7	57.2	
Additionally allowed (%)	39.2	34.6	36.8	
Generously allowed (%)	4.9	6.7	4.6	
Disallowed (%)	2.9	1.9	1.3	

[1] These figures relate to differences in intermodular angles between the two structures within each ensemble.
[2] The poorer Ramachandran statistics arose due to lack of refinement in aqueous solvent.

Fig. S1

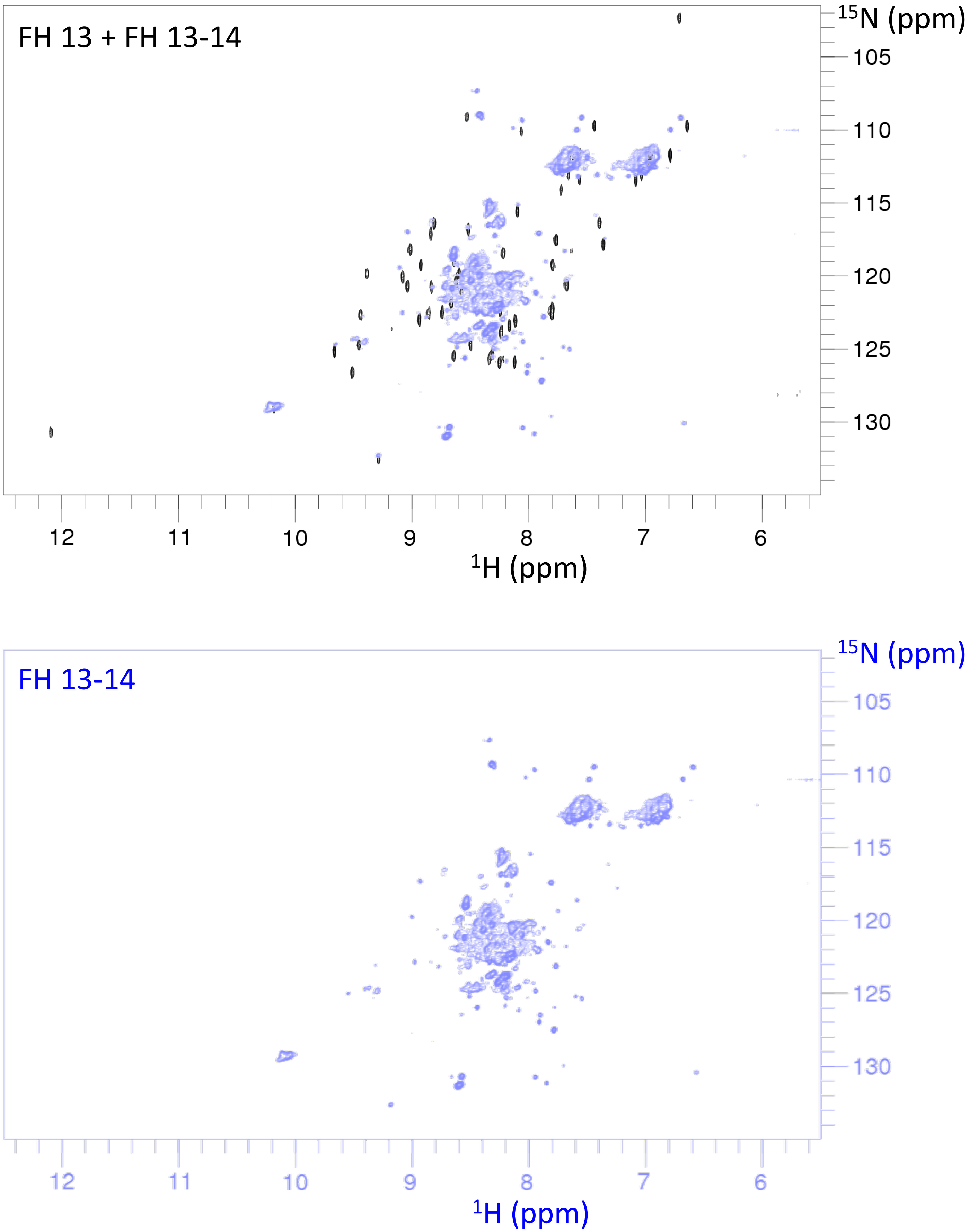

FH 13 + FH 13-14
15N (ppm)
105
110
115
120
125
130
12
11
10
9
8
7
6
1H (ppm)
FH 13-14
15N (ppm)
105
110
115
120
125
130
12
11
10
9
8
7
6
1H (ppm)

Fig. S2

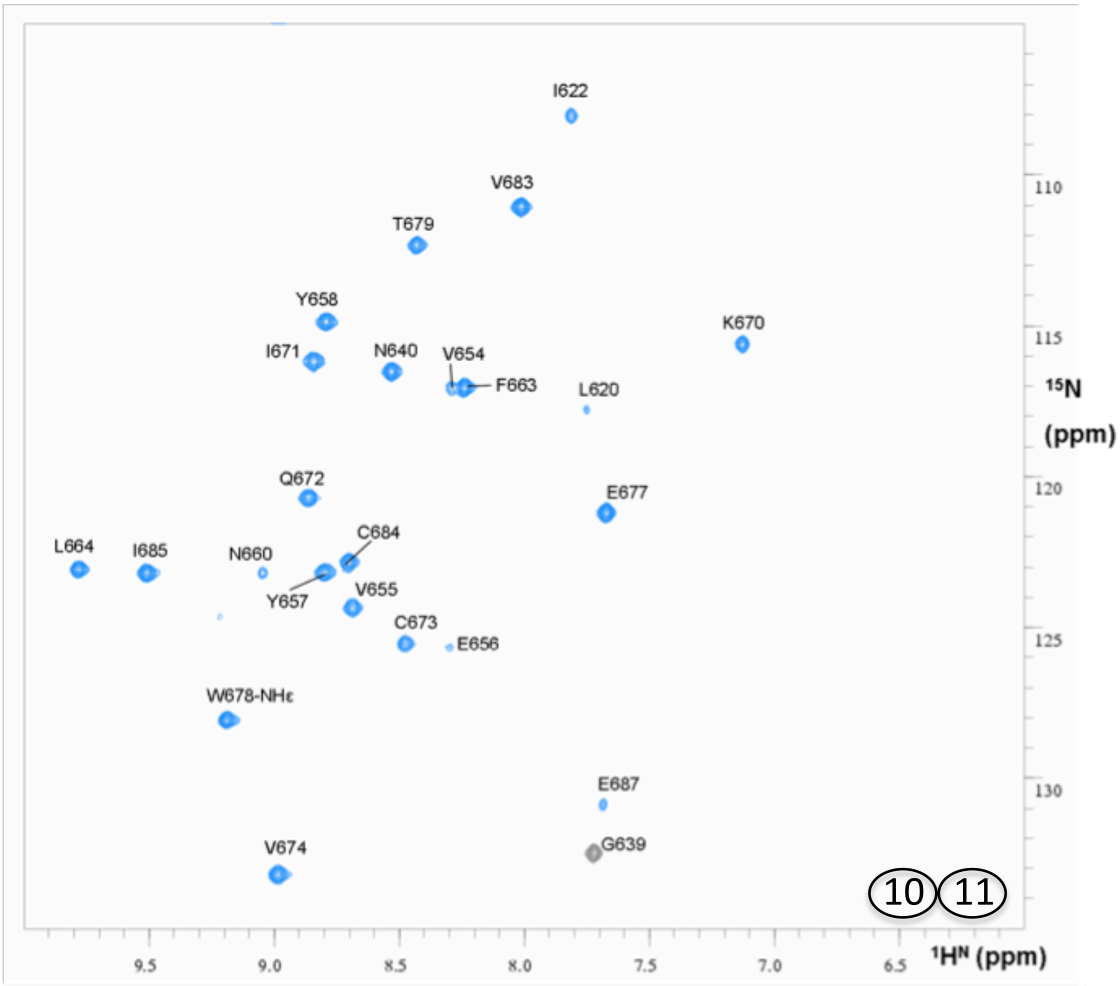

I622
V683
T679
Y658
K670
I671
N640
V654
F663
L620
Q672
E677
L664
I685
N660
C684
Y657
V655
C673
E656
W678-NHε
E687
G639
V674
10 11
9.5
9.0
8.5
8.0
7.5
7.0
6.5
110
115
120
125
130
¹⁵N (ppm)
¹Hᴺ (ppm)

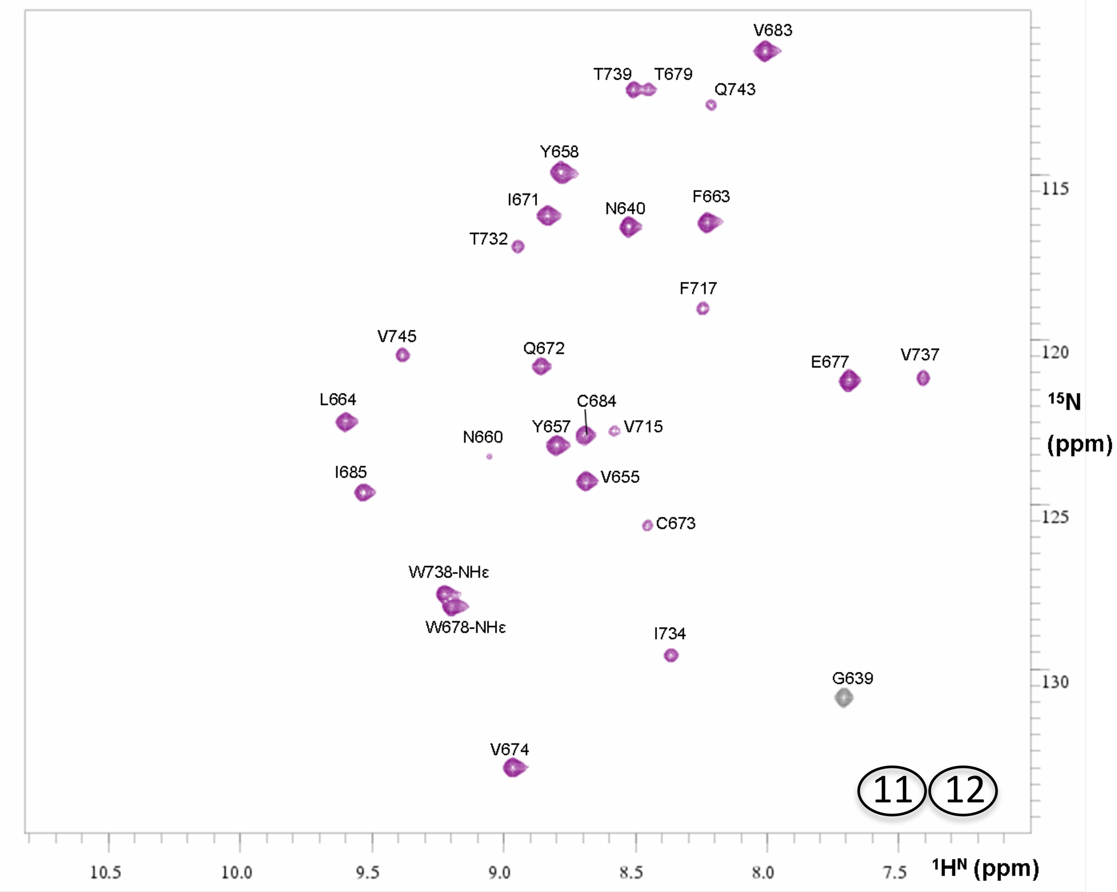

V683
T739
T679
Q743
Y658
I671
N640
F663
T732
F717
V745
Q672
E677
V737
L664
C684
N660
Y657
V715
I685
V655
C673
W738-NHε
W678-NHε
I734
G639
V674
11 12
10.5
10.0
9.5
9.0
8.5
8.0
7.5
115
120
125
130
¹⁵N (ppm)
¹Hᴺ (ppm)

Fig. S3

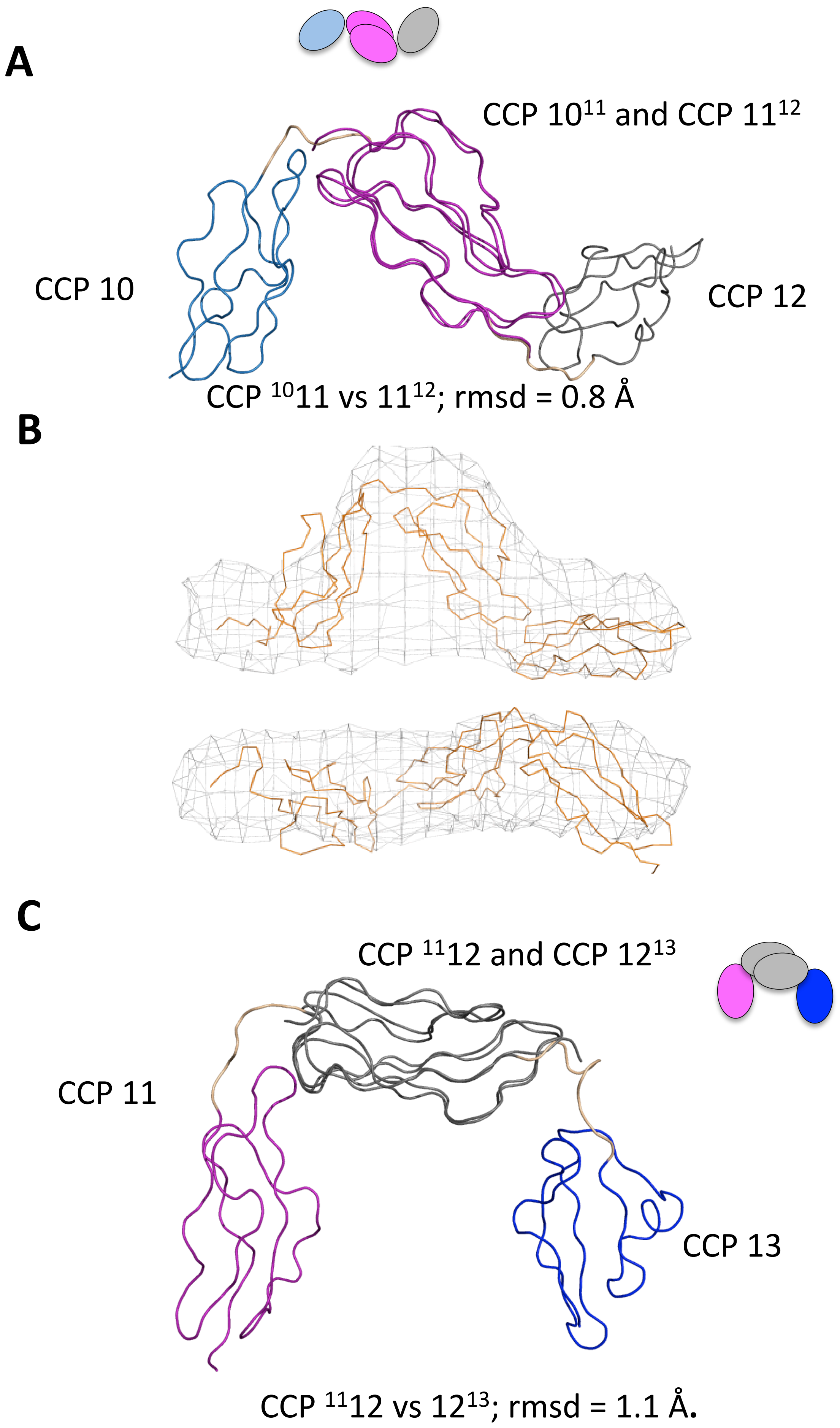

A
CCP 10^{11} and CCP 11^{12}
CCP 10
CCP 12
CCP ^{10}11 vs 11^{12}; rmsd = 0.8 Å
B
C
CCP ^{11}12 and CCP 12^{13}
CCP 11
CCP 13
CCP ^{11}12 vs 12^{13}; rmsd = 1.1 Å.

Fig. S4

```
FH_CCP10     1 ECELPKIDVHLVPDRK-KDQYKVGEVLKFSCKPG-----FTIVGPNSVQCYHF-GLSPDLPICKEQ
MCP_CCP3     1 LCTPPPKIKNGKHTFSEVEVFEYLDAVTYSCDPAPGPDPFSLIGESTIYCGDNSVWSRAAPECKVV
```

A

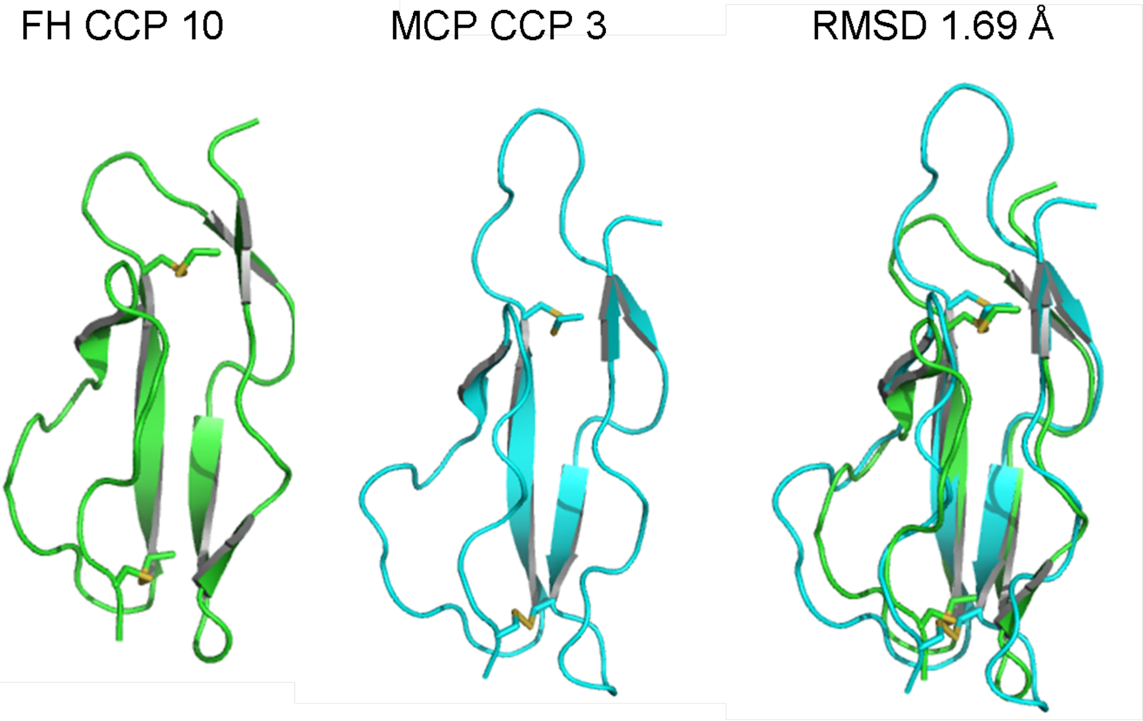


```
FH_CCP12     1 IPELEHGWAQLSSPPYYYGDSVEFNCSESFTMIGHRSITCI-HGVWT--QLPQCVAI
CR2_CCP2     1 EPIVPGGYKIRGSTPYRHGDSVTFACKTNFSMNGNKSVWCQANNMWGPTRLPTCVSI
```

B

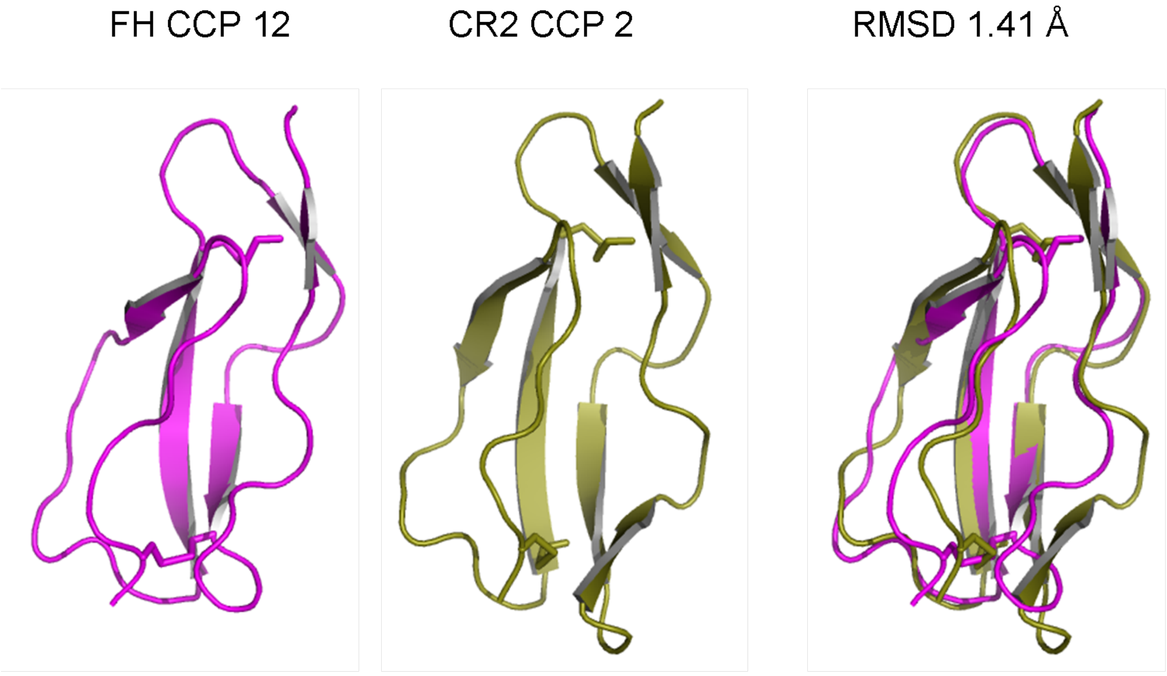

Fig. S4 contd

```
FH_CCP11     1 SCGPPPELLNGNVKEKTKEEYGHSEVVEYYCNPRFLMKGPNKIQCVDGEWTTLPVCIVE
FH_CCP19     1 KCGPPPPIDNGDITSFPLSVYAPASSVEYQCQNLYQLEGNKRITCRNGQWSEPPKCLHP
```

C

FH CCP 11a   FH CCP 19   RMSD 1.18 Å

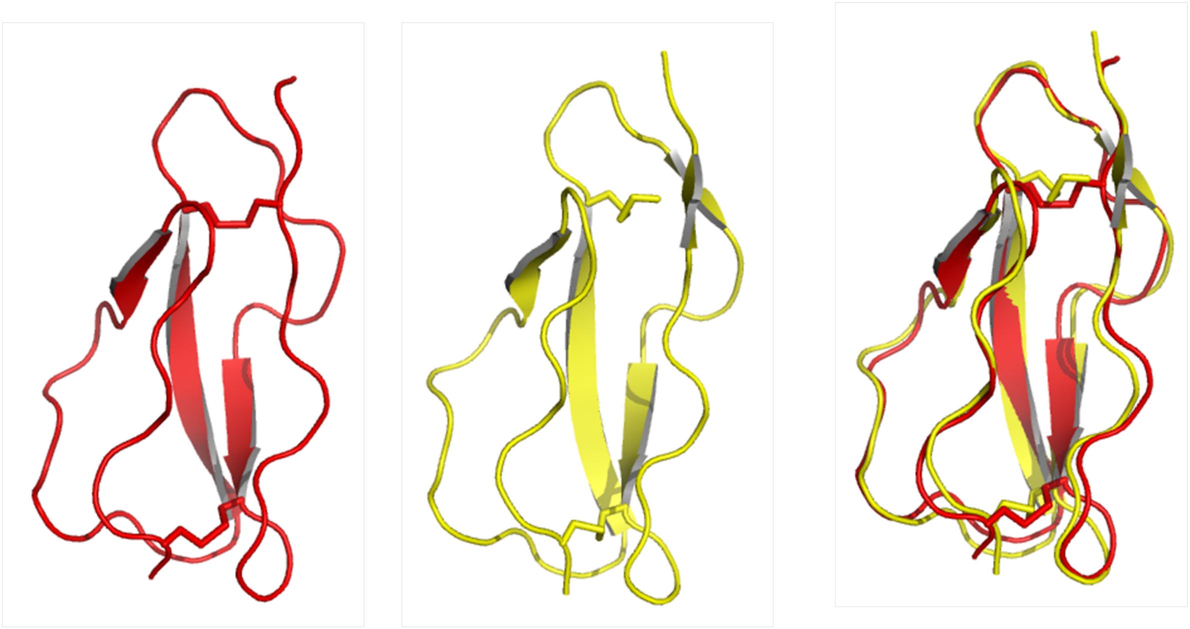

Fig. S5A

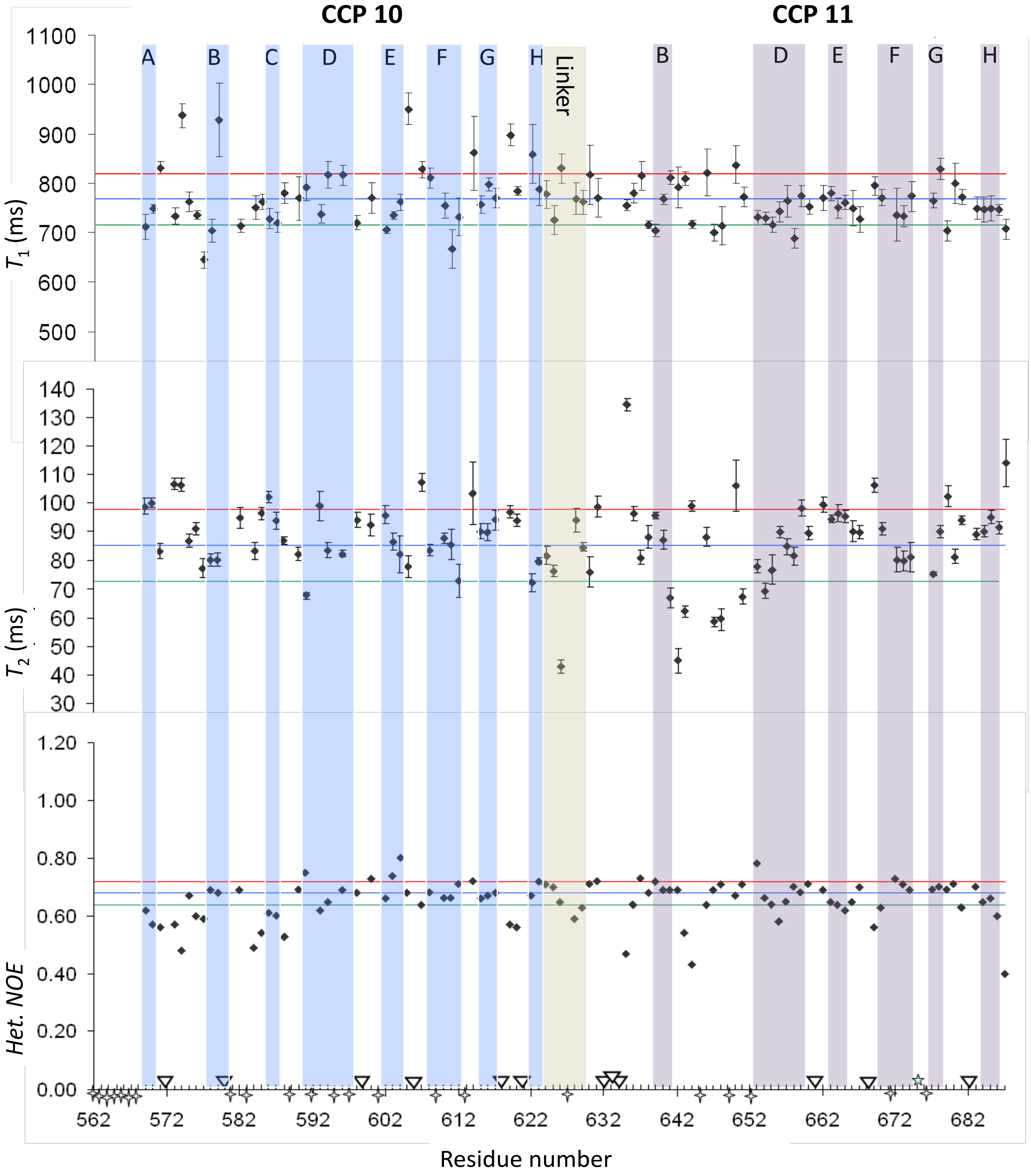
CCP 10
CCP 11
A
B
C
D
E
F
G
H
Linker
B
D
E
F
G
H
1100
1000
900
800
700
600
500
T_1 (ms)
140
130
120
110
100
90
80
70
60
50
40
30
T_2 (ms)
1.20
1.00
0.80
0.60
0.40
0.20
0.00
Het. NOE
562
572
582
592
602
612
622
632
642
652
662
672
682
Residue number

Fig. S5B

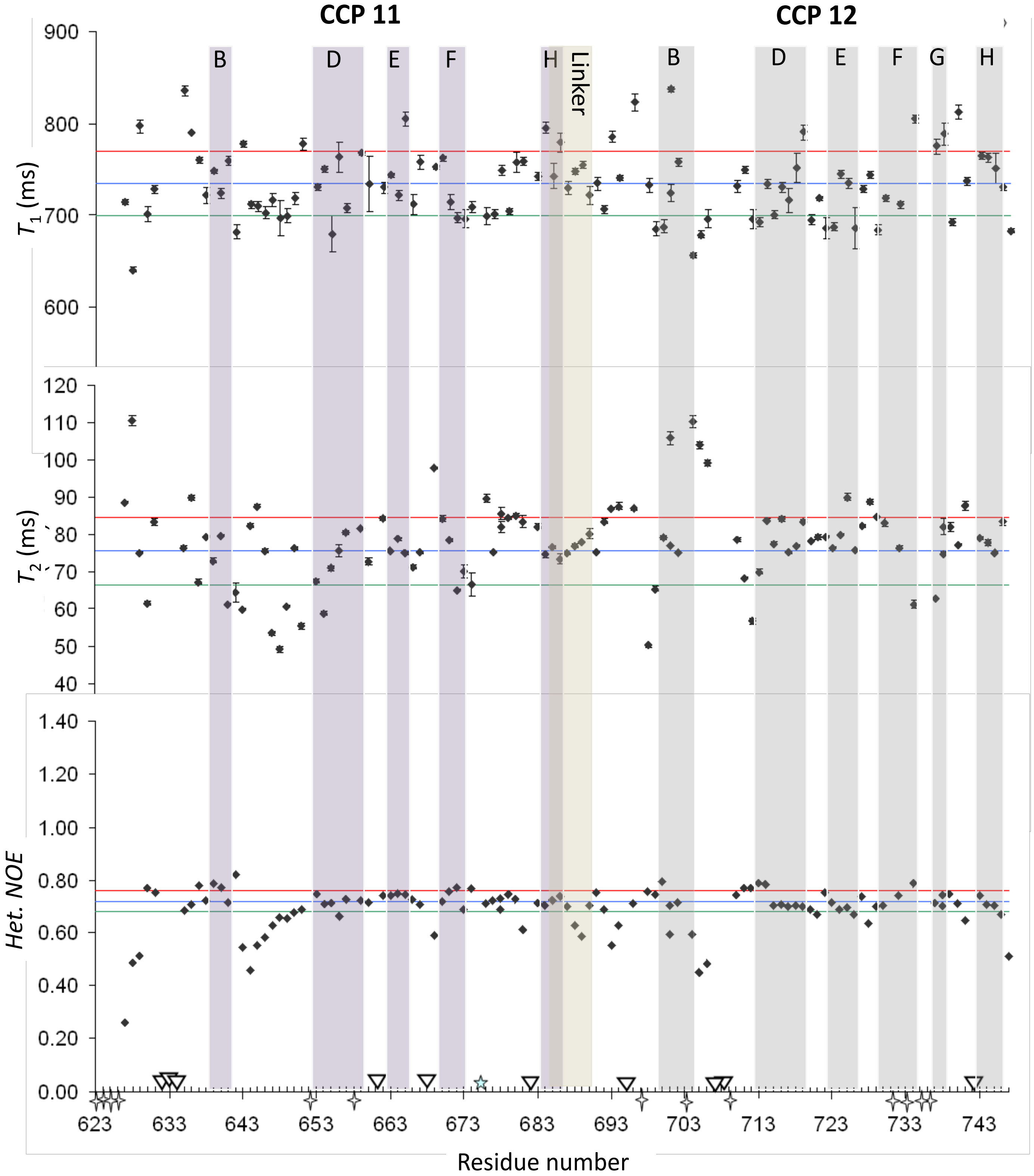

CCP 11
CCP 12
B
D
E
F
H
Linker
B
D
E
F
G
H
T_1 (ms)
T_2 (ms)
Het. NOE
Residue number

Fig. S6

A

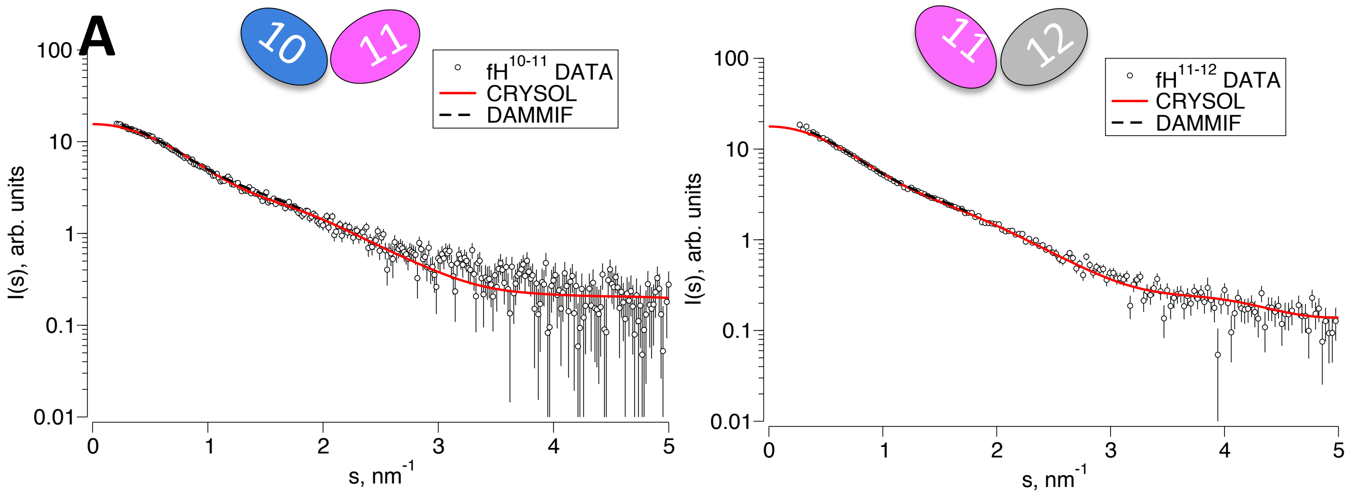
10
11
fH10-11 DATA
CRYSOL
DAMMIF
I(s), arb. units
s, nm-1
11
12
fH11-12 DATA
CRYSOL
DAMMIF
I(s), arb. units
s, nm-1

B

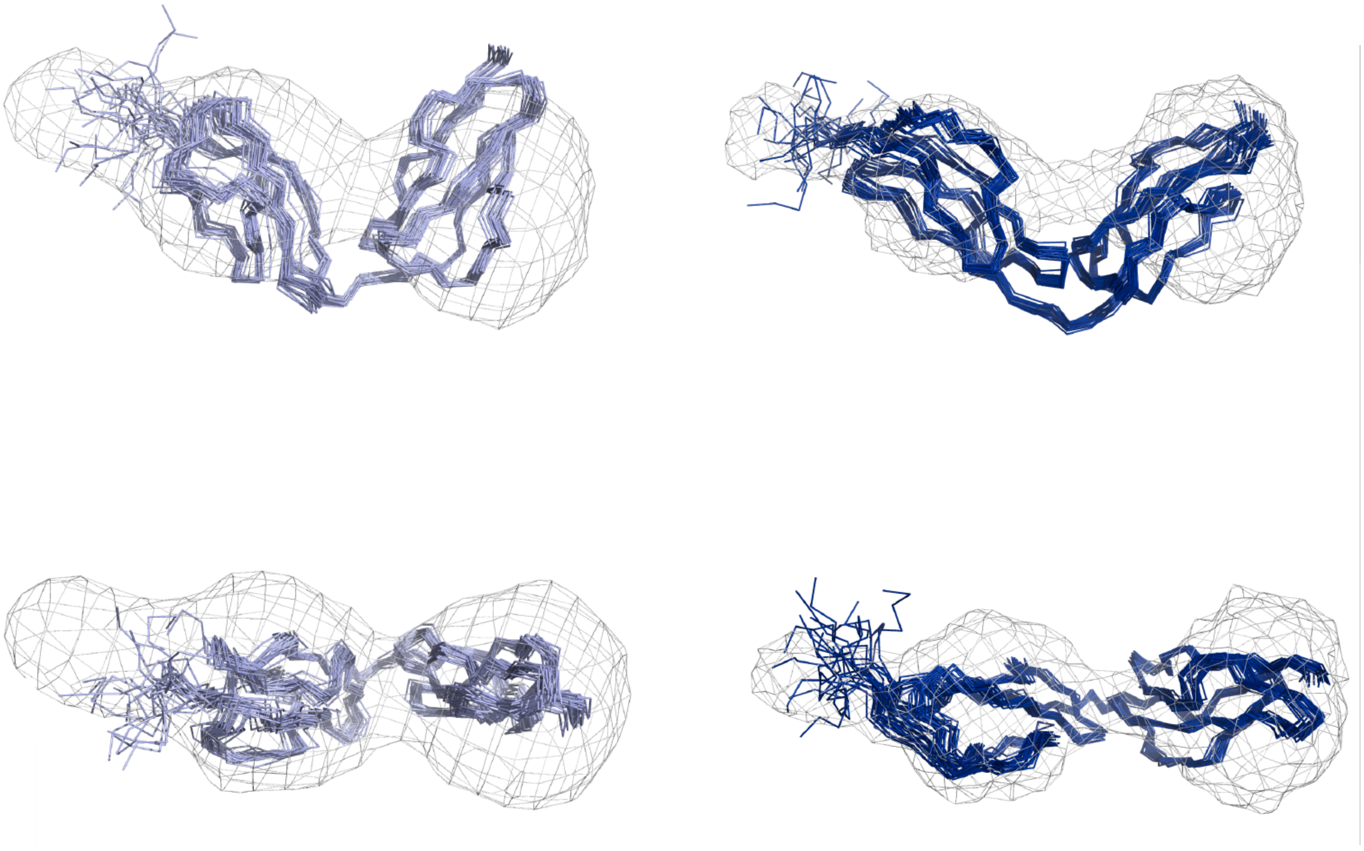

Fig. S7

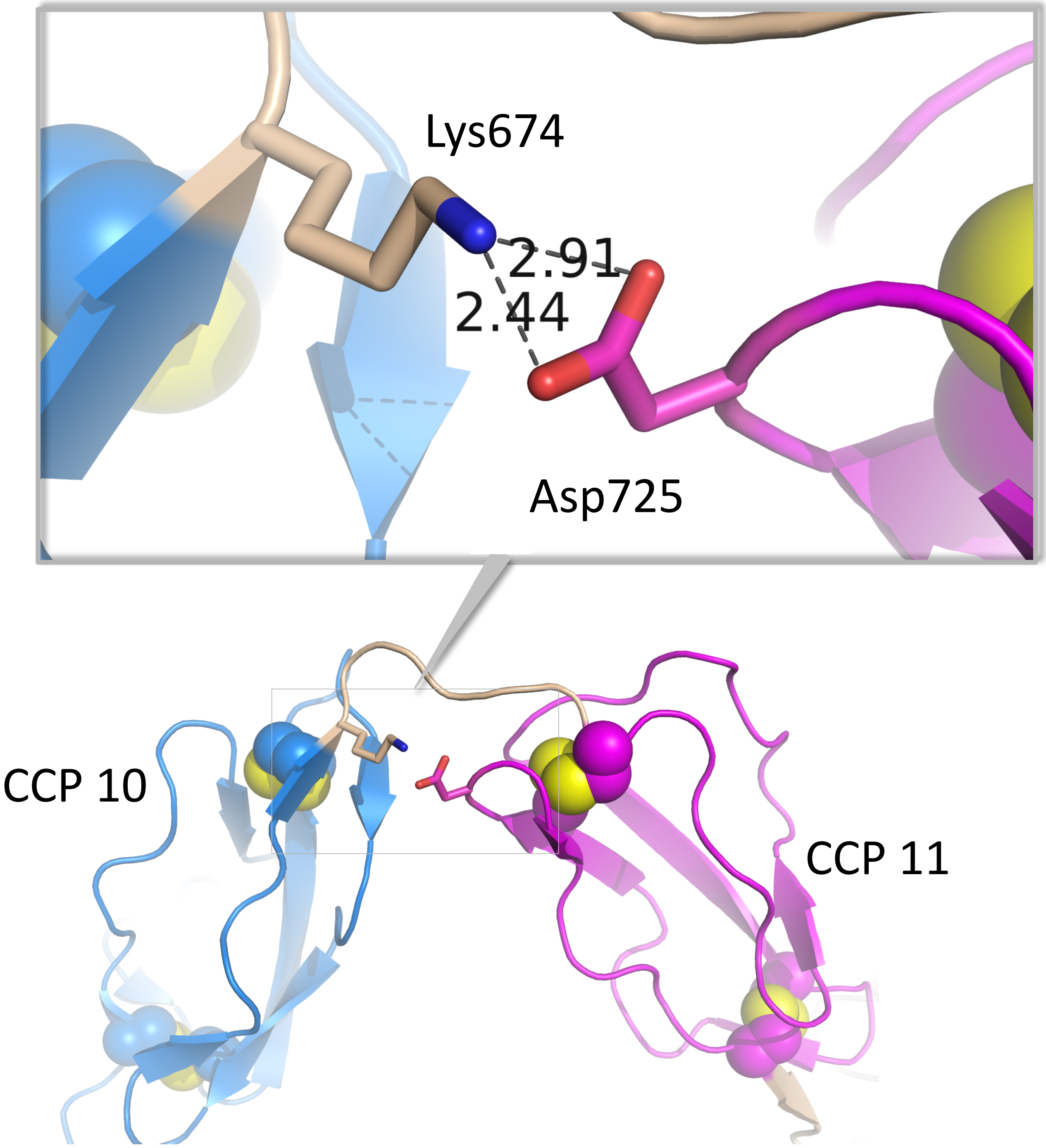

Fig. S8

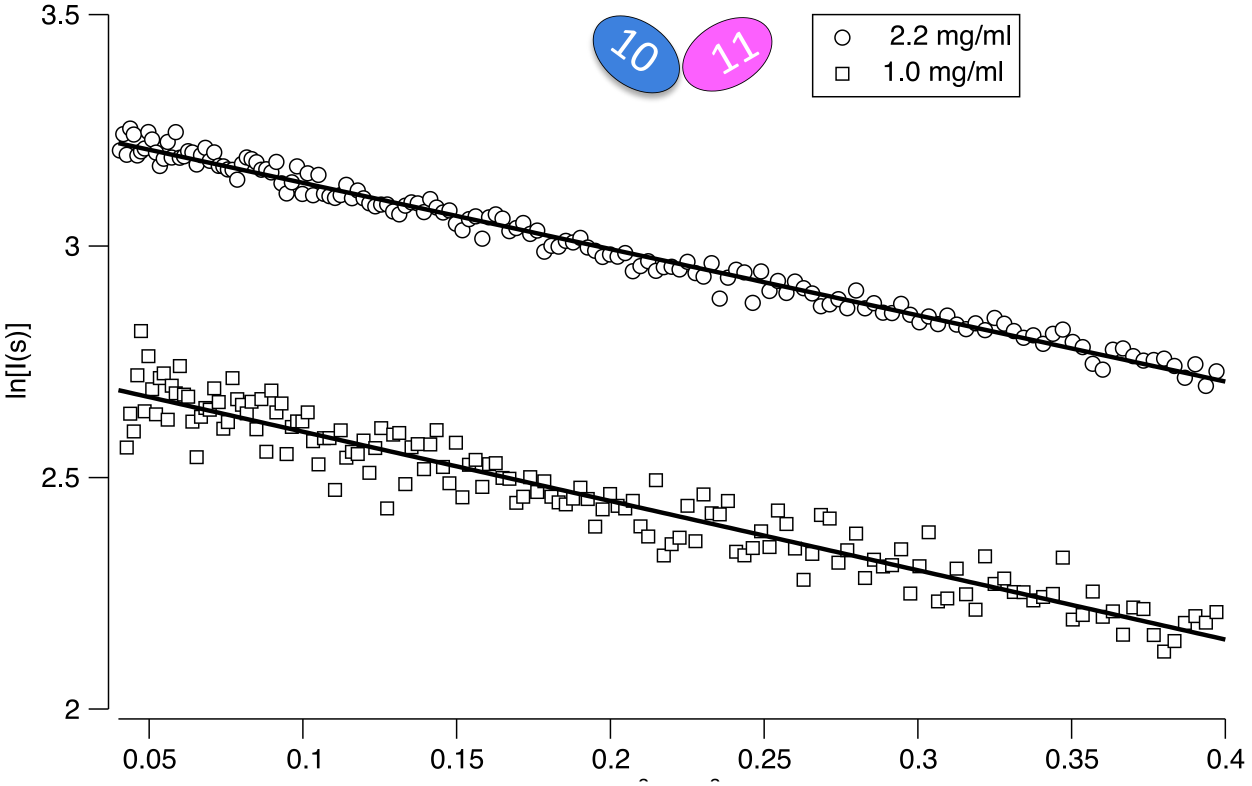

10
11
2.2 mg/ml
1.0 mg/ml
3.5
3
2.5
2
ln[I(s)]
0.05
0.1
0.15
0.2
0.25
0.3
0.35
0.4

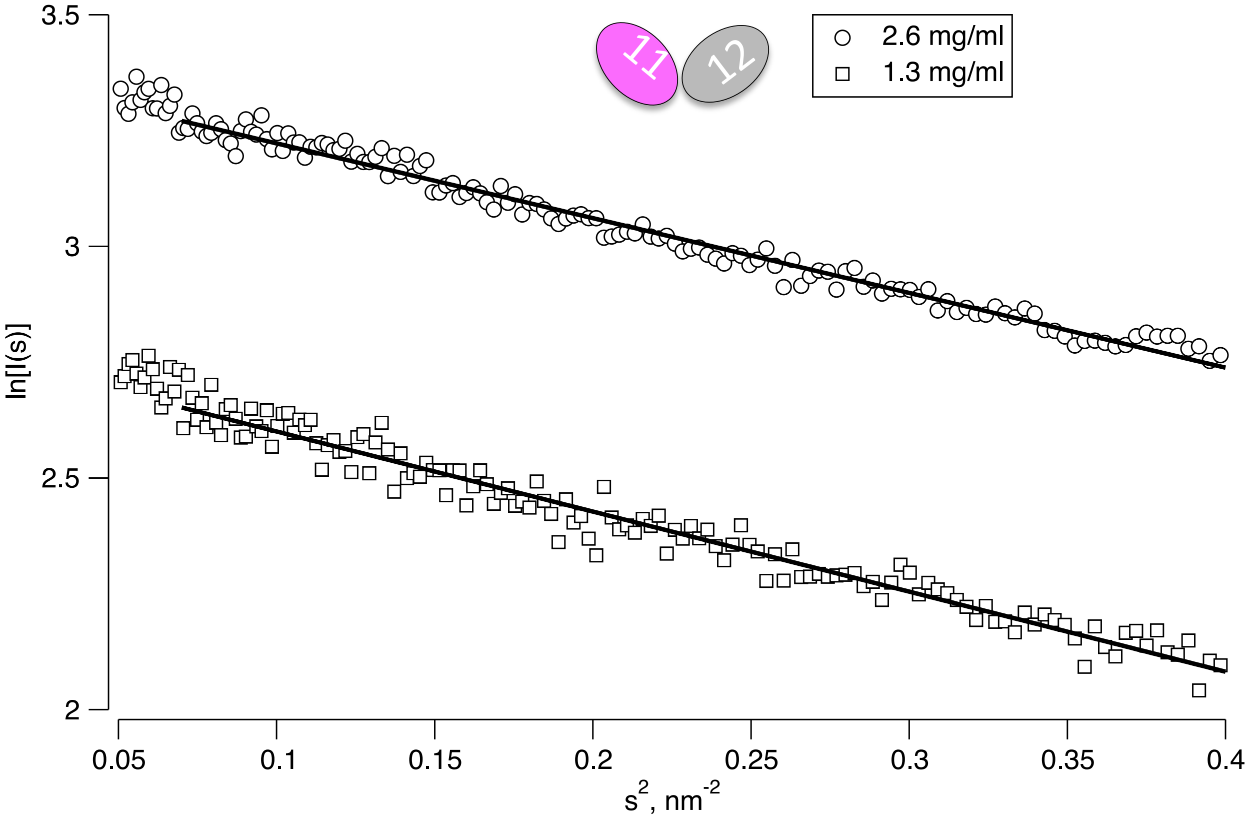

11
12
2.6 mg/ml
1.3 mg/ml
3.5
3
2.5
2
ln[I(s)]
0.05
0.1
0.15
0.2
0.25
0.3
0.35
0.4
s^2, nm^-2
